# Host‐related factors explaining interindividual variability of carotenoid bioavailability and tissue concentrations in humans

**DOI:** 10.1002/mnfr.201600685

**Published:** 2017-02-27

**Authors:** Torsten Bohn, Charles Desmarchelier, Lars O. Dragsted, Charlotte S. Nielsen, Wilhelm Stahl, Ralph Rühl, Jaap Keijer, Patrick Borel

**Affiliations:** ^1^ Luxembourg Institute of Health Strassen Luxembourg; ^2^ NORT, Aix‐Marseille Université, INRA INSERM Marseille France; ^3^ Department of Nutrition, Exercise and Sports University of Copenhagen Frederiksberg C Denmark; ^4^ Institute of Biochemistry and Molecular Biology I Heinrich‐Heine‐University Düsseldorf Düsseldorf Germany; ^5^ Paprika Bioanalytics BT Debrecen Hungary; ^6^ MTA‐DE Public Health Research Group of the Hungarian Academy of Sciences Faculty of Public Health University of Debrecen Debrecen Hungary; ^7^ Human and Animal Physiology Wageningen University Wageningen The Netherlands

**Keywords:** Absorption, Biodistribution, Genetic polymorphisms, Intestine, Macula lutea

## Abstract

Carotenoid dietary intake and their endogenous levels have been associated with a decreased risk of several chronic diseases. There are indications that carotenoid bioavailability depends, in addition to the food matrix, on host factors. These include diseases (e.g. colitis), life‐style habits (e.g. smoking), gender and age, as well as genetic variations including single nucleotide polymorphisms that govern carotenoid metabolism. These are expected to explain interindividual differences that contribute to carotenoid uptake, distribution, metabolism and excretion, and therefore possibly also their association with disease risk. For instance, digestion enzymes fostering micellization (PNLIP, CES), expression of uptake/efflux transporters (SR‐BI, CD36, NPC1L1), cleavage enzymes (BCO1/2), intracellular transporters (FABP2), secretion into chylomicrons (APOB, MTTP), carotenoid metabolism in the blood and liver (LPL, APO C/E, LDLR), and distribution to target tissues such as adipose tissue or macula (GSTP1, StARD3) depend on the activity of these proteins. In addition, human microbiota, e.g. via altering bile‐acid concentrations, may play a role in carotenoid bioavailability. In order to comprehend individual, variable responses to these compounds, an improved knowledge on intra‐/interindividual factors determining carotenoid bioavailability, including tissue distribution, is required. Here, we highlight the current knowledge on factors that may explain such intra‐/interindividual differences.

**Abbreviations**
ADME: absorption, distribution, metabolism and excretion
AMD: age related macular degeneration
*AMY1*: salivary amylase gene 1
ABCA1: ATP binding cassette subfamily A, member 1
ABCG5/G8: ATP binding cassette subfamily G, member 5/8
*ADH7*: Alcohol dehydrogenase 7
ALDH1: aldehyde dehydrogenase 1
APOA, 1–4: apolipoprotein A, 1–4
APOB/C2/E/48: apolipoprotein B/C2/E/48
AUC: area under (plasma/serum concentration‐time) curve
BCO1/2: β‐carotene oxygenase 1/2
CD36: cluster of differentiation 36 molecule
CES1/2: human carboxyl‐esterase 1/2
CETP: cholesteryl ester transfer protein
CLPS: colipase
COBLL1: cordon‐bleu WH2 repeat protein like 1
9CRA: 9‐cis‐retinoic acid
9CDHRA: 9‐*cis*‐13, 14‐dihydro‐retinoic acid
CRISPR/CAS9: clustered regularly interspaced short palindromic repeats/protein‐9 nuclease
CXCL8: C‐X‐C motif chemokine ligand 8
CYP26B1: cytochrome P450 family 26 subfamily B member 1
CYP7A1: bile acid synthetic enzyme
ELOVL2: elongation of very long chain fatty acids like 2
GI: gastro‐intestinal
GPS: protein pathway suppressor
GSTP1: glutathione S‐transferase pi 1
HNF4A: hepatocyte nuclear factor 4, alpha
FABP2/I‐FABP: fatty acid binding protein, intestinal
FGF4/19: fibroblast growth factor 4/19
FOXO1: forkhead box O1
FXR: farnesoid X receptor
IL8: interleukin 8
INSIG2: insulin induced gene 2
IRS1: insulin receptor substrate 1
ISX: intestine specific homeobox
KD: equilibrium dissociation constant
LCAT: lecithin‐cholesterol acyl‐transferase
LDLR: low density lipoprotein receptor
LIPC: lipase C, hepatic type
*LIPF*: gastric lipase
LPL: lipoprotein lipase
LRAT: lecithin‐retinol acyltransferase
LRP1: low density lipoprotein receptor‐related protein 1
LXR: liver X receptor
MC4R: melanocortin 4 receptor
MTTP/MTP: microsomal triglyceride transfer protein/gene
NF‐κB: nuclear factor kappa‐B
NRF2/NFE2L2: nuclear factor (erythroid‐derived 2) like 2
NPC1L1: NPC1 like intracellular cholesterol transporter 1
*PGA3/4/5*: pepsinogen3/4/5
*PGC*: progastricsin
PGC1α: peroxisome proliferator‐activated receptor gamma coactivator 1‐alpha
PKD1L2: polycystin 1 like 2
PLRP2: pancreatic lipase‐related protein‐2
PNLIP: pancreatic lipase
PPAR: peroxisome proliferator‐activated receptor
PXR: pregnane X receptor
RAR: retinoic acid receptor
RBP1/3/4: retinol binding protein 1/3/4
RPE65: retinal pigment epithelium specific protein 65kDa
RSD: relative standard deviation (RSD = SD/mean), equal to CV (coefficient of variation)
RXR: retinoid X receptor
RXRA: retinoid X receptor alpha
SAR1B: secretion associated Ras related GTPase 1B
SR‐BI/SCARB1: scavenger receptor class B member 1, protein/gene
SHP: short heterodimer partner
SNP: single nucleotide polymorphism
SETD7: SET domain containing lysine methyltransferase 7
SLC27A6: solute carrier family 27 (fatty acid transporter), member 6
SOD2: superoxide dismutase 2, mitochondrial
StARD3: StAR related lipid transfer domain containing 3
STRA6: stimulated by retinoic acid gene 6 protein homolog
T2D: type II diabetes mellitus
TCF7L2: transcription factor 7 like 2
TRL: triacylglycerol‐rich lipoprotein fraction
WT: wild‐type

## 1 Introduction

**Figure 1:**
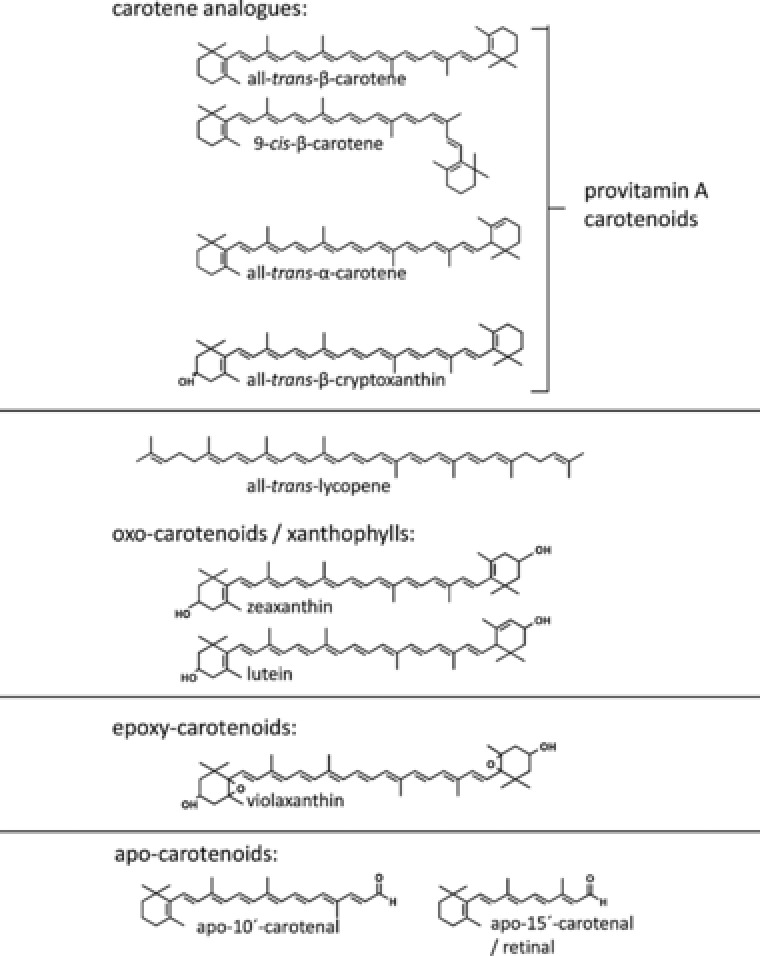
Predominant carotenoids in our diet, common metabolites and nomenclature.

Carotenoids are natural pigments with a C‐30 or C‐40 backbone. They can be produced by most plants, bacteria, and fungi, but not by animals or humans, making diet their sole source. Carotenoids have recently been investigated with much interest, as their dietary intake and endogenous concentrations have been associated with a reduced risk of several chronic diseases. For example, carotenoid intake has been positively associated with a reduced risk of cancer 1, type 2 diabetes mellitus (T2D) 2, cardiovascular diseases 3, and asthma 4, while plasma carotene concentration was shown to be significantly associated with reduced total mortality 5. In addition, some carotenoids, including α‐, β‐carotene and β‐cryptoxanthin (Fig. 1), are vitamin A precursors, constituting the predominant source of vitamin A in most developing countries (up to 90% 6) as well as in Western countries especially with respect to vegetarians. Recently, it has also been suggested that *cis*‐carotenoids are even more beneficial for the prevention of atherosclerosis and T2D than their all‐*trans* isomers 7, 8. Finally, it is now acknowledged that lutein and zeaxanthin play a role in vision by improving contrast sensitivity and visual acuity 9 and participate in the prevention of age‐related macular degeneration 10.

However, several intervention trials with carotenoid supplements have not supported these beneficial associations, or even found negative health effects 11, 12. To explain this discrepancy, it is hypothesized that the food matrix (missing synergistic effects, e.g. with other antioxidants such as polyphenols), a larger array of natural occurring carotenoids compared to single carotenoids in high amounts, presentation in the form of carotenoid supplements (powder, solid matrix) and continuous intake in case of supplements, influence absorption, distribution, metabolism and excretion (ADME), and therefore also their bioactivity. However, it has also been emphasized that ADME‐related factors, including digestion and matrix release, solubilisation in mixed micelles, epithelial uptake in the (small) intestine, and further bio‐distribution, all prerequisites for exerting potential biological effects, can be different between individuals. This likely results in variable blood/tissue concentrations 13, 14, 15. However, blood plasma/serum alone may not constitute the best indicator to assess carotenoid status, and additional methods, such as isotopic labelling, similar as for retinoids 16 or easy accessible compartments such as white blood cells 17 or buccal cells 18, may allow for more insights regarding endogenous carotenoid levels, carotenoid compartments, and turnover 19.

This intra‐ and interindividual variability can be attributed, in addition to dietary habits 20, 21, to host‐related factors (Table 1) including disease state 22, 23, 24, possibly physical activity 25, 26, being overweight/obese 26, alcohol use 25, 27, 28, smoking habits 26, drug intake 29, age 30, and genetic aspects 31, 32. However, the underlying mechanisms for this variability – e.g. lower bioaccessibility, reduced absorption, altered tissue distribution, turnover, and excretion as well as possible interactions of individual carotenoids on absorption and bio‐activation of other carotenoids 33, 34, are only poorly understood. These factors can result in huge variability of carotenoid absorption and circulating plasma levels (Table 2). In a double tracer study 13 with D_6_ β‐carotene (37 μmol), lowest AUC (area under the plasma‐concentration‐time curve, μmol h/L) versus highest AUC were found to be 0.01 and 30.00, respectively. Major host factors influencing carotenoid ADME patterns are likely to include:

- Factors influencing carotenoid release from the food matrix, and their transition from lipid droplets to mixed micelles, i.e. factors impacting bioaccessibility. This includes genes responsible for the expression of digestive enzymes (e.g. gastric lipase, cholesterol esterase, pancreatic lipase, etc.), and bile acid formation aiding in carotenoid micellization 20, 35;
- Factors altering carotenoid uptake into (or efflux out of) the intestinal epithelium. This encompasses uptake/efflux transporters such as scavenger receptor class B member 1 (SR‐BI), cluster of differentiation 36 (CD36), and Niemann‐Pick C1 like intracellular cholesterol transporter 1 (NPC1L1), and perhaps other ATP‐binding cassette (ABC) proteins such as ABCG5/G8, or ABCA1 31, 36, but also small intestinal surface available for absorption;
- Factors contributing to intracellular cleavage, especially BCO1/2 (β‐carotene oxygenase 1/2), responsible for centric/asymmetric cleavage of carotenoids, respectively, producing a variety of retinoids and potential endogenous occurring apocarotenoids 37, 38, 39;
- Factors that impinge on carotenoid intracellular transport in the gut epithelium, i.e. lecithin‐retinol acyltransferase (LRAT) for retinol (and perhaps other β‐carotene cleavage products), and maybe intestinal fatty acid binding protein (FABP2/I‐FABP);
- Factors altering the secretion of carotenoid‐containing chylomicrons into the lymphatic system, such as apolipoprotein B 48 (APOB48), APOAIV, SAR1B (secretion associated Ras related GTPase1B), and microsomal triglyceride transfer protein (MTTP) 38;
- Factors influencing carotenoid transport in the blood plasma/serum, such as their further distribution into lipoproteins, e.g. lipoprotein lipase (LPL), APOA‐I, APOB, APOE and perhaps APOC3, and low density lipoprotein receptor (LDLR) 40; and the liver such as by hepatic lipase (LIPC);
- Deposition of carotenoids in “target tissues”, e.g. the macula (lutein, zeaxanthin), influenced by SR‐BI, glutathione S‐transferase P1 isoform (GSTP1), StAR‐related lipid transfer domain protein 3 (StARD3), BCO1, cholesterol transporters (SR‐BI, ABCA1, ABCG5/8), retinal pigment epithelial‐specific protein (RPE65), elongation of very long chain fatty acids like 2 (ELOVL2), and those involved in visual pigment metabolism 41. However, also deposition in adipocytes, involving e.g. LDLR, could play a role 42;
- Any factor associated with carotenoid catabolism and excretion (in addition to BCO1/2), possibly those involving cytochrome P450 enzymes or the aryl‐hydrocarbon‐receptor 43;
- The microbiota. This may alter e.g. patterns and concentrations of secondary bile acids 44, or possibly carotenoid absorption or degradation patterns 45, though it is not sure if a significant fraction of carotenoids can be absorbed from the colon 46.
- Effects of individual carotenoids on the absorption, binding, transport and bioactivation of other carotenoids as well as selective absorption, binding, transport and bioactivation of individual carotenoids 33.
- Any factor associated with vitamin A/retinoid storage and metabolism.

**Table 1:** Overview of host (non‐dietary) factors proposed to influence (in addition to genetic make‐up and malabsorption diseases of the GI) intra‐and interindividual differences regarding carotenoid ADME

Factor	Type of study	Carotenoids investigated and variability	Reference
Age	Observational, *n =* 400 adults (males, females)	Younger age correlated with lower serum carotenoids	164
	Observational, *n =* 946 postmenopausal women	Lower serum lycopene levels associated with higher age	30
	Observational, *n =* 12500	Lower serum β‐carotene levels with older age	272
	Review	Higher plasma carotenoid levels with older age	273
Alcohol	Observational, *n =* 2895 women	No consistent effect of alcohol consumption on plasma levels of α‐carotene, β‐carotene, β‐cryptoxanthin, and lutein‐zeaxanthin	26
	Observational, *n =* 194 men	Negative correlations of plasma levels of lycopene, β‐carotene, β‐cryptoxanthin (not lutein) with units of alcohol/day, Spearman's rank correlation: ‐0.27 to ‐0.51	25
	Observational, *n =* 1198 subjects	Higher alcohol consumption related to higher plasma lycopene (ca. 20%), no effect on α‐and β‐carotene, lutein, and β‐cryptoxanthin	28
	Intervention, *n =* 12 healthy men	Consumption of wine, beer, or spirits for 3 weeks reduced plasma β‐carotene by 15%, no effect on lycopene, lutein, zeaxanthin, ‐cryptoxanthin, and α‐carotene	27
	Observational, *n =* 400 adults (male, female)	Higher alcohol consumption correlated with lower serum carotenoids	164
	Observational, *n =* 12500 adults (male, female)	Lower β‐carotene levels with alcohol consumption	272
Asthma	Observational, women with (n = 84) & without asthma (n = 47)	Higher plasma total‐carotenoids in women with asthma	253
Body weight, BMI	Observational, *n =* 2895 women	Obese women had lower plasma levels of α‐carotene, β‐carotene, β‐cryptoxanthin, and lutein‐zeaxanthin, by ca. 10%, compared to normal‐weight women. Plasma lycopene was higher by 10%	26
	Observational, *n =* 194 men	Negative correlation of BMI with serum lutein but not lycopene, β‐carotene, β‐cryptoxanthin, R: ‐0.12 (Spearman rank correlation)	25
	Observational, *n =* 400 adults (males, females)	Higher BMI associated with lower α‐and β‐carotene and xanthophyll serum levels	164
	Observational, *n =* 946 postmenopausal women	Higher BMI correlated with lower plasma lycopene levels	30
	Observational, *n =* 600 healthy adults	Higher abdominal obesity related to lower serum carotenoid levels (α‐,β‐carotene, canthaxanthin	274
	Observational, *n =* 55 women	Similar total adipocyte β‐carotene content in lean and obese, β‐carotene concentration reduced in obese	194
Gender	Observational, *n =* 12,500 adults (male, female)	Women had higher serum β‐carotene levels than men	272
Helicobacter pylori infection	Observational, *n =* 49 anemic patients (male, female)	Lower gastric mucosal β‐carotene reported with increased H. pylori infection (though no effect on plasma β‐carotene levels)	22, 275
HIV	Observational, *n =* 1669 women	Lower serum β‐carotene levels in HIV subjects	276
Hyperthyroidism	Observational, *n =* 36 patients	Lower serum β‐carotene in subjects with hyperthyroidism compared to hypo‐and euthyroidism	254
Low zinc status	Intervention, *n =* 12 males	Supplementation with zinc (20 mg/d) improved plasma carotenoid concentration	112
	Observational, *n =* 400 women HIV positive	Lower serum β‐carotene associated with markers of disease progression, univariate regression, R: – 0.083‐0.244	24
	Observational, *n =* 1665 men and women, healthy and diabetic	20% lower plasma β‐carotene levels in diabetes subjects compared to healthy ones	277
Blood lipids, cholesterol	Observational, *n =* 400 adults (male, female)	Higher non‐HDL cholesterol associated with lower serum carotenoids	164
	Observational, *n =* 12,500 (male, female)	Higher total cholesterol and lower triglycerides associated with higher β‐carotene in serum	272
Drug intake	Intervention, *n =* 8 volunteers	Intake of simvastatin (lipid‐lowering drug), 40 mg/day for 8 weeks, reduced plasma levels of carotenes (lycopene, α‐and β‐carotene) and xanthophylls (β‐cryptoxanthin, lutein), by 5 and 21%, respectively	29
	Intervention trial, *n =* 6 patients (1 male, 5 female)	Intake of orlistat (lipid‐lowering drug) decreased levels of α‐, and β‐carotene in plasma	278
	Intervention trial, *n =* 228 obese subjects (male, female)	Intake of orlistat (lipid‐lowering drug) decreased β‐carotene levels in plasma	279
Malaria	Observational, *n =* 100 malaria and 50 control children (boys, girls)	Lower serum concentration of all major carotenoids compared to control	280
Menstrual cycle	Intervention trial, *n =* 9 women	Lower plasma carotenoids during early than late follicular phase	166
Microbiota	Observational, *n =* 25 subjects (males, females)	*Collinsella* spp. were reduced in subjects with atherosclerosis. These subjects had lower β‐carotene in serum and the metagenome showed lower phytoene‐dehydrogenase.	133
Physical activity	Observational, *n =* 2895 women	Exercising women (>1 time/week) had higher levels of α‐carotene, β‐carotene, β‐cryptoxanthin, and lutein‐zeaxanthin, by ca. 5–10%, compared to normal‐weight individuals without exercise. No effect on lycopene	26
	Observational, *n =* 194 men	Positive correlation of plasma levels of β‐cryptoxanthin and lutein with physical activity, rho: 0.12 to 0.17	25
Race/Ethnicity	Observational, *n =* 4231 children and adolescents (male, female)	African American children had higher β‐cryptoxanthin, lutein, zeaxanthin, & lycopene serum concentrations but lower α‐carotene conc. than white children (not adjusted for dietary intake)	281
	Observational, *n =* 285 healthy adolescents (male, female)	African‐American participants had lower serum concentrations of α‐carotene, but higher conc. of lutein + zeaxanthin compared with Caucasians (not adjusted for dietary intake)	282
Smoking	Observational, *n =* 194 men	No relation of smoking to plasma levels of lycopene, β‐carotene, β‐cryptoxanthin, lutein	25
	Observational, *n =* 1198 adults (male, female)	Increased smoking related to lower plasma lycopene, α‐ and β‐carotene, lutein, β‐cryptoxanthin (ca. 10–30%), total carotenoids ca. 50% lower	283
	Observational, *n =* 400 adults (male, female)	Smoking correlated with lower serum carotenoids	164
	Observational, *n =* 12,500 adults (male, female)	Smoking correlated with lower β‐carotene blood concentrations (not adjusted for dietary intake)	272

**Table 2:** Studies investigating the variability of carotenoids in blood and target tissues, following intervention trials and observational studies

Study design	Carotenoid(s)	Tissue/Compartment	Variability	Reference
Observational: 901adult subjects during 4 years (male, female)	Lycopene, lutein, α‐carotene, β‐carotene, β‐cryptoxanthin	Plasma conc.	Intraindividual variance: Lutein/zeaxanthin: 20.7% β‐carotene: 21.0% α ‐carotene: 21.9% Lycopene: 35.0% β‐cryptoxanthin: 27.1% Interindividual variance: Lutein/zeaxanthin: 70.5% β‐carotene: 70.7% α ‐carotene: 67.5% Lycopene: 61.0% β‐cryptoxanthin: 66.6%	284
Observational: 381 adult women, 4‐month intervals, 4 visits	Lutein	Plasma conc.	Interindividual: 47% RSD Intraindividual: 44% of interind. variation	243
	β‐carotene	Plasma conc.	Interindividual: 80%RSD Intraindividual: 34% of interind. variation	
	Lycopene	Plasma conc.	Interindividual: 41% RSD Intraindividual: as interind. variation	
Observational: 21 adult subjects over 1 year (male, female), 6 measurements	β‐carotene	Plasma conc.	Interindividual: 100% RSD Intraindividual: 21% of interind. variation	244
	Lycopene	Plasma conc.	Interindividual: 42% Intraindividual: 72% of interind. variation	
Double stable isotope to 11 healthy men (37μmol β‐carotene)	β‐carotene	Plasma AUC	Interindividual: 137%%RSD 300 fold differences in AUC dose response observed	13
Intervention: 8 adult subjects (4 males, 4 females), 0.5 μmol/kg bw.	β‐carotene	Plasma AUC	Intraindividual: 68% RSD	159
Intervention: 8 adult subjects, 0.5 μmol/kg bw.	Lutein	Plasma AUC	Intraindividual: 43% RSD	
Administration of isotopically labelled lycopene (10.2 mg) to 8 subjects (4 males, 4 females)	Lycopene	Absorption % based on plasma AUC	Interindividual: 504% RSD for all trans‐lycopene	15
Administration of lycopene (10‐120 mg) in a tomato beverage (5 male adults)	Lycopene	Absorption (%)	Interindividual: 77% RSD for highest dose, 53% RSD for lowest dose	160
Administration of soup, juice or tablets to 6 adult males (ca. 20 mg lycopene)	Lycopene	Plasma AUC	Interindividual: <28% RSD	285
Feeding trial (5 weeks, 9 mg lutein/d) to young males	Lutein	Plasma conc.	Interindividual: ca. 70% RSD	286
Administration of tomato puree, spinach (12 mg β‐carotene, 8 mg lutein), and pills containing β‐carotene and lutein (20 young females)	Lycopene β‐carotene Lutein	Plasma Plasma TRL AUC	Interindividual: 40 % RSD Interindividual: 40% RSD Interindividual: ca. 45% RSD from spinach	147
Administration of tomato puree to 33 adult men (0.4 mg β‐carotene)	β‐carotene	TRL AUC	Interindividual: 105% RSD	32
β‐arotene in oil within a meal (120 mg), 80 males	β‐carotene	TRL AUC	Interindividual: 61% RSD	287
Administration of tomato sauce (17 mg β‐carotene) to 12 adults (male, female)	β‐carotene	TRL AUC	Interindividual: 64% RSD	149
Administration of tomato puree, 33 adult men, 10 mg lycopene	Lycopene	TRL AUC	Interindividual: 70% RSD	73
Administration of tomato juice to *n =* 18 adults (male, female). Ca. 22 mg lycopene	Lycopene	TRL AUC	Interindividual: <67% RSD	288
Administration of tomato sauce to 12 adults (male, female). Ca. 47 mg lycopene	Lycopene	TRL AUC	Interindividual fractional absorption: 2.4% (RSD: 83%)	148
Administration of tomato preparations to 30 adult men, 25 mg lycopene	Lycopene	TRL AUC	Interindividual: 96% RSD for tomato paste	289
Administration of supplement (s) or tomato puree (tp) to 39 healthy men, ca. 5 mg lutein	Lutein	TRL AUC	Interindividual: RSD of 75% and 137% for s and tp, respectively	14
Administration of salad and avocado oil to *n =* 11 healthy subjects (male, female). 12 mg β‐carotene, 6 mg lutein, 7 mg α ‐carotene	Lutein β‐carotene α‐carotene	TRL AUC TRL AUC TRL AUC	Interindividual RSD: 54% 69% 82%	290
Administration of tomato puree, carrots, spinach, intragastrically to 10 adult males. 10 mg of each carotenoid	Lycopene Lutein β‐carotene	Duodenum, micellar phase	Interindividual RSD: 32% 23% 20%	66
Observational, 20 ceased subjects (male, female), 0.4 months‐86 years of age	Lycopene β‐carotene α‐carotene Lutein β‐cryptoxanthin	Liver	Interindividual RSD: 123% 124% 149% 123% 243%	173
Observational, 15 ceased adults (male, female), 0.4 months‐86 years of age	Lycopene β‐carotene α‐carotene Lutein β‐cryptoxanthin	Kidney	Interindividual RSD: 100% 132% 132% 234% 234%	173
Observational, 13 ceased adults (male, female), 0.4 months‐86 years of age	Lycopene β‐carotene α‐carotene Lutein β‐cryptoxanthin	Lung	Interindividual: 196% RSD 125% 117% 136% 180%	173
Intervention: 30 mg β‐carotene/d for 43 days in patients with adenomatous polyps (*n =* 7, male, female)	Lutein/zeaxanthin β‐cryptoxanthin Lycopene α‐carotene β‐carotene	Colon	Interindividual RSD: 71% 126% 68% 76% 66%	291

Thus, as individual responses can depend on many varying factors, it is paramount to understand these and their influence on the biological variability of carotenoid ADME. In this review, it is aimed to highlight known host‐related factors that predispose for variations in carotenoid metabolism, such as genetic factors (e.g. single nucleotide polymorphisms (SNPs)), though additional ones (disease state, body weight, smoking, physical activity etc.) are also briefly reviewed. The manuscript structure is oriented around the metabolic path of carotenoids, from digestion (chapter 2) to intestinal absorption (chapter 3) and further transport to the liver (chapter 4) and distribution to target tissues (chapter 5) to storage and excretion related pathways (chapter 6). Searched databased included Pubmed and Scopus, for all years, in English language, employing the following search terms (abstract and title) to start with: “Human* AND (lutein OR lycopene OR xanthophyll OR carotene*) AND (bioavailab* OR pharmacokinetic* OR kinetic* OR absorption OR postprandial OR metabol* OR microb* OR microflora OR biliary OR enterhohepatic* OR chylomicron OR plasma OR tissue OR metabolism OR enterocyte OR lipoproteins OR transporters OR Single nucleotide polymorphism* OR genetic varia* OR SNP OR cleavage OR enzym* OR intestine) AND (intra* OR inter*) NOT (drug‐interaction OR in‐vitro)”, though additional literature following the primary search results were surveyed.

## 2 Host factors influencing digestion aspects – from matrix release to bioaccessibility

### 2.1 General aspects and oral phase of digestion

Bioavailability of carotenoids depends on their bioaccessibility, i.e. the release from the food matrix and subsequent availability for absorption. As carotenoids are apolar, with octanol/water partition coefficients of 8–12 47, their incorporation into mixed micelles is necessary prior to their cellular uptake, which is assumed to take place predominantly in the small intestine.

Mastication during oral digestion results in enhanced surface area and the breakdown into smaller particles. In addition, saliva appears to contain some lipase activity 48, though not necessarily lingual lipase (a triacylglycerol‐lipase, EC 3.1.1.3) 49, 50 (Table 3). As exposure in the oral cavity is rather short (usually less than 1 min), the enzymatic effect on carotenoid bioavailability is presumably small, though smaller particle size has been related to improved carotenoid bioavailability 51. To our knowledge, no mutagenesis on or polymorphisms with effects on oral lipases and lipid digestion has been reported to date.

**Table 3:** Host factors influencing carotenoid release from food matrix and bioaccessibility

Phase of digestion	Factor	Study description	Carotenoids investigated	Possible role in bioavailability	Reference
Oral	Lingual lipase, other lipase	No data available	n/n	Low	n/n
	α‐amylase	No data available	n/n	Low	n/n
Gastric	Non‐dietary phospholipids/ mucin	No data available	n/n	Low compared to dietary phospholipids	n/n
	Gastric lipase (GL)	GL from fungi (Rhizopus oryzae), pH optimum 5–9, in‐vitro	β‐carotene	No effect of gastric lipase detected	64
	Pepsin	No effect in in vitro trials	Lutein, β‐carotene, lycopene	Presumable low effect in most carotenoid rich foods	57, 58
	Pepsin	Tomato puree in vitro	Lycopene	Enhancing effect on lycopene micellization	59
	pH	Digestion of spinach, in vitro	β‐carotene, lutein	Presumably negligible^b^, though extreme pH may facilitate degradation	55, 292
Duodenum	Pancreatic lipase	Digestion of spinach, in vitro	β‐carotene, lutein, zeaxanthin	Low micellization (<5% original conc.) without pancreatin^a^	57
		Digestion of carrots+spinach+ tomato, in vitro	Total carotenoids	Micellization drop to 50% without pancreatin	58
	Pancreatic amylase	Intake of amylase inhibitor ascarbose reduced vit. A levels in blood	Only vit. A	Low	293
	Pancreatic proteases, PLRP2	No data available	n/n	Low	n/n
	Pancreatic colipase	Tomato puree, in vitro digestion	Lycopene	Reduced intestinal recovery without colipase, no effect on micellization	59
	Carboxyl‐esterase	Digestion of wolfberry, pepper, squash in vitro	Zeaxanthin‐esters	Enhanced xanthophyll bioavailability	77
	Bile salts	Digestion of spinach, in vitro	β‐carotene, lutein, zeaxanthin	Micellization drop to 30% original conc. without bile salts	57
	Bile salts	Digestion of carrots+spinach+ tomato, in vitro	Total carotenoids	Low micellization (<2% original conc.) without bile extract	58
Colon	Microbiota	Lower circulating carotenoids in subjects with higher *Collinsella* and atherosclerosis	β‐carotene	Unclear	133
	Microbiota	Higher liver storage of α ‐and β‐carotene in germ‐free rats	α‐, β‐carotene	Prevention of breakdown products? Transit time? Bile‐salts?	45

n/n: no data available.

a though containing also other enzymes, pancreatic lipase is presumably the enzyme most important for carotenoid digestion.

b except for epoxy‐carotenoids (violaxanthin, neoxanthin).

As salivary alpha‐amylase (EC 3.2.1.1) participates in the break‐down of starch, food matrices rich in both starch and carotenoids, such as sweet potato, may be influenced by alterations in alpha‐amylase levels. It has been reported that in populations traditionally exposed to high levels of starch, more copies of the salivary amylase gene (*AMY1)* and higher enzyme levels were found 52, though its influence on the digestion of carotenoids has never been investigated.

### 2.2 Gastric phase of digestion

In the stomach, the primary digestion enzymes include pepsin (3.4.23.1) and gastric lipase (3.1.1.3), though orally secreted lipases may still be active. In addition, a small amount of phospholipids 53 is released from the mucus layer 54, aiding in the emulsification of lipophilic constituents. The pH may have an influence, as low pH can result in the degradation of epoxy‐carotenoids (e.g. violaxanthin, neoxanthin), resulting in epoxide‐furanoid transitions 55. Human gastric pH is influenced mostly by meal, with a complex meal increasing the pH from initially 2 to 3–5, though interindividual differences in fasting pH exist 35.

A few native foods are rich in both proteins and carotenoids, including egg yolk, salmon, and some types of cheese, and protein digestion could contribute to the release of carotenoids. In addition, (partly) digested proteins may aid in emulsifying carotenoids 51. Expression of pepsin has been reported to depend on the pepsinogen genes *PGA3*, *PGA4*, *PGA5*, and progastricsin (*PGC)* 56. However, varying the amount of pepsin in *in vitro* trials did not appear to have measurable effects on carotenoid bioaccessibility from leafy vegetables 57, and at least for such and similar sources, variations in pepsin are not expected to contribute to plasma level variability. Similarly, using a test meal composed of meat, carrots, spinach and tomato paste, gastric digestion (in vitro) had no significant influence on carotenoid bioaccessibility 58, suggesting rather small effects on carotenoid bioavailability at this step, though in these trials, gastric lipase was not involved. By contrast, Periago et al. 59 reported a positive effect of pepsin on lycopene micellization from a puree in vitro. It is possible that for this very apolar carotenoid, protein degradation products added to the emulsifying effect, or aided in matrix breakdown.

The genes related to the production and secretion of mucus containing phospholipids, which could aid in the emulsification process, are not clearly identified. Concentration variations of phospholipids between 0.03 and 0.6 mM have been reported, 60 and may be expected to have some influence on carotenoid micellization. However, their influence and strengths of effect are unknown and would also be superseded by dietary phospholipids, which are expected to play a more important role. This would be true especially following ingestion of lipid‐rich meals (a mean intake of 2–8 g/d of phosphatidylcholine has been reported 61, which would translate into ca. 8 mM (if taken within 1 out of 3 major meals per day, and dissolved in 1 L gastric fluid).

Gastric lipase, encoded by the *LIPF* (lipase F, gastric type) gene 62 and secreted by gastric chief cells, can digest up to 25% of the ingested lipids 35. It thus could be expected to influence the accumulation of carotenoids in lipid droplets, and their degradation, important for the following transition of carotenoids from lipid droplets to mixed micelles. This occurs mostly in the small intestine. Unfortunately, gastric lipase cannot, at present, be studied in vitro, due to the unavailability of human gastric lipase. Other sources, such as those from fungi, have different cleavage kinetics, differing in their pH optimum and also the type sequence of cleavage 63. Rabbit lipase would be an interesting option, but is not commercially available. Some, such as lipase from the fungus *Rhizopus oryzae* have been tested (cleavage optimum pH 5–9), though no significant improvement in bioaccessibility was found 64.

### 2.3 Small intestinal phase of digestion

The most crucial step influencing carotenoid bioacessibility is the small intestinal phase. Here, micellization occurs or is completed, following the secretion of bile salts, in addition to pancreatic lipase, and additional enzymes (pancreatic amylase, nucleosidases, trypsinogen, chymotrypsinogen, carboxypeptidase, elastases, phospholipases, and carboxyl ester lipase). Bile salts aid in the emulsification process and formation and stability of the mixed micelles, while pancreatic lipase produces free fatty acids and monoglycerides, fostering emulsification. Thus, it can be expected that modifications of both bile‐acid and pancreatic lipase secretions have strong effects on the micellization of carotenoids, a pre‐requisite for their diffusion to the unstirred water layer prior to absorption 65. This has been confirmed by several in vitro studies, where micellization and resulting bioaccessibility was very much compromised when either bile salts or pancreatic lipase were missing. Without bile, bioaccessibility of total carotenoids fell to 30%, and without pancreatic lipase or both, to below 5% of their original value 57. Similar strong effect were found by Garret et al. 58, where total carotenoid micellization dropped below 5% of the original values without bile salts. The effect of pancreatin was less drastic (reduction by approximately 50%), possibly due to differences between test meals. In order to study factors influencing lycopene bioaccessibility, tomato puree was digested under various conditions, testing among other factors gastric pH, gastric digestion time, pepsin concentration, intestinal pH, pancreatin concentration, bile salt concentration, colipase addition and intestinal digestion time 59. It was found that only pepsin positively influenced micellization, while olive oil had a slightly negative effect, likely due to entrapment of lycopene by non‐hydrolysed olive oil.

Following intragastric in vivo administration of carotenoid rich meals, duodenal fluid was aspirated, and micellization determined 66. Variability between subjects’ micellization efficacy (fractional bioaccessibility) was considerably lower compared to plasma or triacylglycerol‐rich lipoprotein (TRL) carotenoid variability following interventions, being 20, 23, and 32%, respectively for β‐carotene, lutein and lycopene, vs. typically 50–80% for plasma, though variations between studies can be considerable (Table 3). This may point out that, although enzyme or bile salt concentrations surely play a role in interindividual variation, an additional and about equal portion of variability is added during and after absorption.

Bile acid production by the liver is governed by a variety of genes, involving for instance bile acid synthetic enzyme (*CYP7A1*), activators of *CYP7A1* expression such as HNF4α (hepatocyte nuclear factor 4 alpha, encoded by *HNF4A*), and PGC1α (encoded by *PPARGC1A*), repressors of *CYP7A1* (farnesoid X receptor (FXR, encoded by *NR1H4*)), short heterodimer partner (SHP, encoded by *NR0B2*), G protein pathway suppressor 2 (GPS 2, encoded by *GPS2*), pregnane X receptor (PXR, encoded by *NR1I2*), fibroblast growth factor 19 (FGF19; encoded by *FGF19*), fibroblast growth factor receptor 4 (FGFR4; encoded by *FGFR4*), klotho B (encoded by *KLB*), and forkhead box O1 (FOXO1; encoded by *FOXO1*) 67, however, their role in carotenoid absorption and tissue variability has not been examined.

At least three lipases are secreted from the pancreas, including pancreatic triglyceride lipase (encoded by *PNLIP*), which is the most abundant lipase (producing sn2‐monoacylglycerol and free fatty acids), but also two homologues, pancreatic lipase‐related proteins 1 (not apparently active regarding lipolysis) and 2 (PLRP1 and PLRP2) 68. PLRP2 possess a broader substrate specificity, also cleaving, unlike PNLIP, phospholipids and galactolipids. The frequency of a SNP in the *PLRP2* gene (rs4751995) has been associated with populations historically consuming a diet rich in cereals, and may have repercussions on lipid digestion 69.

Though pancreatic triglyceride lipase activity is usually reduced by bile‐salts, this effect is offset by colipase, also secreted by the pancreas 70. Formation of colipase preprotein is regulated by the *CLPS* gene, and mice deficient for *CLPS* showed lower survival and weight gain on a high‐fat diet, suggesting the inability to cope with lipids on a high fat diet 71. A polymorphism for the gene encoding procolipase has been related to lipid metabolism and diabetes risk 72, and would be an interesting candidate also regarding carotenoid metabolism. In a recent study, a SNP in *PNLIP* (rs11197742) was found in a combination of SNPs associated with chylomicron secretion of lycopene 73, although its contribution was rather low and did not reach statistical significance when investigated individually (*p* = 0.086). Several SNPs in *PNLIP* have been reported in children, and the latter was related to altered plasma lipoprotein and total cholesterol concentrations 74, as well as with lycopene bioavailability (Table 4).

**Table 4:** List of SNPs known, or speculated, to influence carotenoid metabolism

Aspect of bioavailability	Gene	SNP	Carotenoid/other	Function	Reference
Digestion	*PNLIP*	rs11197742	Lycopene^c^	Pancreatic lipase	73
		96A/C^a^ exon 3			74
		486C/T exon 6			74
		1359C/T exon 13	Plasma lipoproteins		74
	*CLPS*	Arg92Cys (rs370885215)	Cholesterol, apolipoproteins^d^	Colipase	72
	*LIPF*	unknown	unknown	Gastric lipase	46
Absorption	*SCARB1* f	Intron‐5	β‐carotene^d^	Transporter	103
		Allele A, exon 1	β‐cryptoxanthin^d^		103
		Allele T, exon 8	β‐cryptoxanthin^d^		103
		rs11057820	Lutein^e^		294
		rs11057841	Lutein^d^		294
		rs10773109	Lutein^d^		294
		rs11057830	Lutein^d^		294
		rs11608336	Lutein^d^		294
		rs12581963	Lutein^d^		294
		rs10846744	Lutein/zeax^d^		295
		rs11057841	Lycopene^d^		296
		rs61932577	β‐carotene, α‐carotene^d^		84
		rs5888	β‐cryptoxanthin		84
	*CD36* f	rs4112274	Lycopene^c^	Transporter	73
		rs1524598	Lutein/zeaxanthin^d^		295
		rs1761667	Lutein/zeaxanthin^d^		215
		rs13230419	Lutein/zeaxanthin^d^		215
		rs1761667	Lutein/zeaxanthin^e^		215
		rs1984112	β‐cryptoxanthin^d^		84
		rs1761667	β‐cryptoxanthin^d^		84
		rs7755	β‐cryptoxanthin^d^		84
		rs1984112	α‐carotene^d^		84
		rs1761667	α‐carotene^d^		84
		rs1527479	α‐carotene^d^		84
	*NPC1L1*	rs17725246	Lycopene^c^	Transporter	73
		rs217430	Lutein/zeax^d^		295
		rs217428	Lutein^d^ ?^h^		215
		rs17655652	Lutein^d^?		215
		rs217434	Lutein^d^?		215
	*ABCG5*	rs2278357	β‐carotene^c^	Transporter	32
		rs10205816	Lutein/zeaxanthin^d^		295
	*ABCG8*	rs13405698	Lutein/zeaxanthin^d^		295
		rs4953028	Lutein/zeaxanthin^d^		295
		rs4148211	Lutein^d^ ?		215
		rs4148217	Lutein^d^ ?		215
		rs6544718	Lutein^d^ ?		215
	*ABCG2*	rs17731631	Lutein^c^	Transporter	14
		rs6532059	Lutein^c^		14
		rs1871744	Lycopene^c^		73
	*ABCA1*	rs2791952	β‐carotene^c^, lycopene^c^	Transporter	32, 73
		rs1331924	Lycopene^c^		73
		rs10991408	β‐carotene^c^		32
		rs3887137	β‐carotene^c^, lycopene^c^		32, 73
		rs390253	Lutein^c^		14
		rs4149316	Lutein^c^, lycopene^c^		73
		rs4149299	Lycopene^c^		
		rs9919066	Lutein^c^		14
		rs2020926	Lutein^c^		14
		rs2274873	Lutein/zeaxanthin^d^		295
		rs1331924	Lutein/zeaxanthin^d^		295
	*ABCB1*	rs10248420	Lycopene^c^		73
		rs10280101	Lycopene^c^		73
	*ISX* g	rs137252	Lutein^c^	Regulates BCO1	14
		rs5749706	Lutein^c^	Expression	14
		rs137269	Lutein^c^		14
		rs137238	Lutein^c^		14
		rs5755368	β‐carotene^c^, lutein^c^		14, 32
		rs202313	β‐carotene^c^		32
		rs16994824	β‐carotene^c^		32
		rs2056983	Lycopene^c^		73
Intracellular cleavage	*BCO1*	rs7196470	β‐carotene^c^	Cleavage enzyme	32
		promotor	β‐carotene^d^		107
		rs11645428	Lutein/zeaxanthin^d^		295
		rs6564851	Lutein/zeaxanthin^d^		295
		rs7500996	Lutein/zeaxanthin^d^		295
		rs6564851	β‐carotene, α‐carotene, lycopene, zeaxanthin, lutein^d^		32, 104
		rs4889286	β‐carotene^d^		297
		rs12934922	β‐carotene^d^		297
		rs4889293	α‐carotene^d^		297
		rs4889286	α‐carotene^d^		297
		rs12918164	β‐cryptoxanthin^d^		297
		rs4889293	β‐cryptoxanthin^d^		297
		rs56389940	Lutein/zeaxanthin^d^		297
		rs10048138	Lutein/zeaxanthin^d^		297
		rs7501331	Lutein^d^^,^ e		215
		rs12934922	β‐carotene^d^		298
		rs7501331	β‐carotene^d^		298
		rs12934922	β‐carotene^d^		298
	*BCO2*	rs12796114	Association with AMD	Cleavage enzyme	295
		rs2250417	Association with AMD		295
Intracellular transport (gut epithelium) and other functions	*ELOVL2*	rs9468304	β‐carotene^c^, lutein^c^	Fatty acid elongase, precursor membrane	14, 32
			Lycopene^c^	Lipids	73
		rs3798709	β‐carotene^c^, lutein^c^, lycopene^c^		14, 32, 73
		rs911196	β‐carotene^c^, lycopene^c^		73
	*INSIG2*	rs17006621	Lutein^c^, lycopene^c^	Sterol binding	14
	*I‐FABP*	IFABP‐Thr	Lycopene^d^	Fatty acid transport	103
	*SLC27A6*	rs10053477	Lycopene^c^	Fatty acid transport	73
Chylomicron secretion	*MTP*	rs17029213	Lutein^c^	Triglyceride	14
		rs17029173	Lycopene^c^	Transporter	73
		rs1032355	Lycopene^c^		73
		rs745075	Lycopene^c^		73
Blood, liver metabolism, lipoprotein distribution	*LPL*	rs7821631	Lutein^c^	Lipoprotein lipase	14
		rs10096561	Lutein^c^		14
		rs1441778	Lutein^c^		14
		rs7841189	Lycopene^c^		73
		rs7005359	Lycopene^c^		73
		rs17482753	Lycopene^c^		73
		X447 allele	Lutein, β‐carotene, α‐carotene, β‐cryptoxanthin^b^		162
	*APOA1*	rs2070665	Lutein^c^	Protein of HDL	14
	*APOA4*	Ser‐347	Lycopene^d^	Chylomicron protein	103
	*APOE*	*ɛ4*	AMD	Chylomicron protein	295
	*APOB*	rs1042031	β‐carotene^c^, lycopene^c^	Protein of LDL, VLDL, chylomicrons	32, 73
		rs4643493	β‐carotene^c^		32
		rs35364714	β‐carotene^c^		32
		rs2854725	Lutein^c^		14
		516	β‐carotene^d^		103
		516	Lycopene^d^		103
	*LDLR*	rs6511720	Tocopherol	Lipoprotein receptor	104
	*LIPC*	rs1869138	β‐carotene^c^	Hepatic lipase	32
		rs11857380	β‐carotene^c^		32
		rs12185072	β‐carotene^c^		32
		rs12591216	Lutein^c^		14
		rs12593880	Lutein^c^		14
		rs8035357	Lycopene^c^		73
		rs12914035	Lycopene^c^		73
		rs493258	Zeaxanthin^d^		299
		rs493258	Lutein^d^		299
		HL C‐480T	α‐,β‐carotene		103
	*CYP26B1*	rs2241057	Retinol	Degradation of retinol	182
	*CETP*	rs708272	Lutein/zeaxanthin^d^	Cholesteryl and perhaps carotenoids ester transfer	295
Tissue	*GSTP1*	Pi (isoform)	Lutein/zeaxanthin	Uptake into retina	300
incorporation	*STARD3*	rs9892427	Lutein/zeaxanthin^d^	Lipid transfer, binding to retina	295
	*RPE65*	rs12139131	β‐carotene^c^		32
		rs4926340	β‐carotene^c^		32
		rs1924546	Lutein^c^		14
		rs12744671	Lutein/zeaxanthin^d^		295
Other functions	*SOD2*	rs2501175	β‐carotene^c^	Antioxidant enzyme	32
		rs9365046	Lycopene^c^		73
	*COBLL1*	rs3769877	Lutein^c^	Insulin metabolism	14
	*CXCL8*	rs1247620	β ‐carotene^c^	IL‐8 precursor	32
		rs1358594	β ‐carotene^c^		32
		rs6834586	β‐carotene^c^		32
	*TCF7L2*	rs946199	β‐carotene^c^	Transcription factor related to diabetes	32
	*PKD1L2*	rs8043708	β‐carotene^c^	Related to pore channels?	32
		rs12596941	Lutein^c^	Ion channel?	14
		rs935933	Lycopene^c^		73
	*MC4R*	rs11873337	Lutein^c^	Obesity	14
	*IRS1*	rs2178704	Lutein^c^	Signal transduction	14
		rs1316328	Lutein^c^		14
	*SETD7*	rs7680948	Lycopene^d^	Insulin metabolism, inflammation	296

a base‐pairs: A: adenine, C: cytosine, T: thymine, G: guanine.

b in animals, not humans.

c Measured by chylomicron response.

d Measured by plasma levels.

e Related to AMD, Measured as macula pigment optical density (MPOD).

f also involved in uptake in other tissues

g Intestine Specific Homeobox.

h Question mark indicating assumed influence.

Carboxyl‐ester lipase (CEL), also termed cholesterol‐esterase, typically cleaves cholesterol esters in the gut, and its ability to cleave carotenoid esters, such as of lutein, present in many leafy vegetables, has been controversially discussed 75. At least five types of CEL are known, though human carboxylesterases CES1 and CES2 may play the most important role during digestion 76. These are situated on the gut mucosa (brush border enzymes), and have shown to cleave carotenoid esters 77. Its origin (pancreatic vs. enterocyte) remains somewhat unclear. However, this cleavage is expected to influence bioavailability, as the more apolar esters are characterized by lower micellization efficiency and absorption than the cleaved carotenoids 78. In fact, in plasma and circulating chylomicrons, free xanthophylls are almost exclusively found, suggesting that cleavage is in fact quite complete 79, though reduced absorption of the esters could play a role. A number of SNPs have been described in humans for CES1 and CES2 80, though not in relation to carotenoid or lipophilic phytochemical/micronutrient metabolism.

Certain diseases such as pancreatitis may also result in lower secretion of digestion enzymes 81. Also during older age reduction of lipid absorption has been reported, perhaps also due to reduced epithelial surface 82, which may thus be expected to correlate with lower carotenoid absorption, as suggested by some, though not all studies (Table 1).

## 3 Host factors determining aspects of intestinal absorption

### 3.1 Factors influencing cellular uptake and cleavage

Following their extraction from the food matrix and incorporation, at least in part, into mixed micelles, carotenoids are taken up by enterocytes. This process is not only passive, as previously thought 83, and several apical membrane proteins have been shown to facilitate carotenoid uptake 36. SR‐BI, encoded by *SCARB1*, is involved in the uptake of β‐carotene 84, 85, lutein 86 and lycopene 87. CD36 facilitates β‐carotene 84 uptake and could facilitate lycopene uptake 88, while NPC1L1 participates in the uptake of lutein 89. All of these proteins have SNPs in their encoding genes associated with carotenoid plasma concentrations (Table 4), and their contribution to carotenoid uptake has been confirmed in cellular models (e.g. human Caco‐2 cell line), but also in models employing transfected kidney (HEK) cells. After enterocyte uptake, carotenoids can be metabolized by BCO1 90 and BCO2 39. BCO1 catalyses the oxidative cleavage of provitamin A carotenoids (chiefly β‐carotene, α‐carotene, β‐cryptoxanthin), apo‐carotenals, and lycopene, but not that of lutein 91. BCO1 is presumably the main cleaving‐enzyme for β‐carotene 92. Lycopene was suggested to be predominantly cleaved by BCO2 93, while recently lycopene cleavage by BCO1 was also reported 94. However, until now no lycopene derived BCO1‐products were determined 95, 96 and were only postulated 97, 98. BCO2 has also been shown to be involved in lutein metabolism 99. Most β‐carotene conversion (>70%) is thought to occur in the intestine; by using stable isotope techniques it was estimated that about 20–30% occurs after absorption 100, contributing to overall vitamin A homeostasis. In addition, a controlled temporal and spatial conversion of carotenoids to bioactive retinoids is also of physiological importance, indicated by a specific pattern of BCO1 expression in various tissues 101. This expression is linked to RAR‐mediated signaling 39, 102.

The involvement of several proteins in the intestinal absorption of carotenoids (apical uptake) suggests that variations in the genes encoding these proteins could modulate carotenoid absorption efficiency. This has been confirmed in an association study by Borel *et al*. 103 where the influence of candidate SNPs of genes involved in lipid metabolism on the fasting blood concentration of several carotenoids was investigated. More specifically, SNPs in *SCARB1* were associated with β‐carotene but not with lycopene concentrations. These SNPs explained differences in β‐carotene plasma concentrations by up to 50%. Several additional SNPs have meanwhile been identified, including several in *BCO1* in genome‐wide association studies 31, 104, 105. Three recent studies have reported associations of combinations of SNPs involved in interindividual variability of the bioavailability of lutein 14, lycopene 73 and β‐carotene 32, employing a candidate gene approach in postprandial studies. In these, plasma‐TRL carotenoids, representing newly absorbed carotenoids, were measured in healthy male adults. These combinations were associated with 73, 72, and 69% of the interindividual variability of the bioavailability of lutein, lycopene and β‐carotene, respectively. While some SNPs were located in genes expressed in other tissues or were closely involved in plasma‐TRL metabolism, others were involved with carotenoid transport or metabolism at the enterocyte level. These included *ABCA1*, *ABCG5*, *BCMO1*, *CD36*, *ELOVL2*, and *ISX* (intestine specific homeobox). Interestingly, one SNP in *ELOVL2* (rs9468304) was very strongly associated with all three phenotypes, possibly due to the inhibitory effect of eicosapentaenoic acid, which is further elongated to docosapentaenoic acid and docosahexaenoic acid by ELOVL2, on carotenoid absorption, as has been shown with β‐carotene 106.

### 3.2 Influence of nutritional status

Host vitamin A status has been linked with β‐carotene absorption variability. Lobo *et al*. 107 demonstrated that the intestinal transcription factor ISX acts as a repressor of *SCARBI* and *BCO1* expression following retinoic acid induction. This mechanism is thought to serve as a negative feedback loop regulating retinal and further retinoic acid, retinyl esters and retinol status through modulation of provitamin A carotenoid absorption and cleavage efficiencies. Interestingly, the same team has reported the existence of an SNP in the ISX binding site in the *BCO1* promoter (rs6564851) which was associated with decreased conversion rates by 50% and increased fasting blood levels of β‐carotene 108.

Though the mechanisms are not fully elucidated, low iron status was suggested to interact with retinol homeostasis, resulting in decreased mobilization of liver vitamin A and thus low serum concentrations 109, possibly involving altered BCO1 activity 110. Also a low zinc status appears to reduce β‐carotene absorption from the gut 111, perhaps as phospholipase A2 can bind zinc and may be more active. These effects were confirmed in human studies, where supplementation with iron and zinc following a vitamin A deficient diet improved retinol and carotenoid plasma appearance, respectively 112. Also low protein status appears to hinder conversion of β‐carotene to vitamin A, contributing to carotenoid variability 113.

BCO1 and BCO2 were also described to be controlled by peroxisome proliferator‐activated receptor (PPAR) – retinoid X receptor (RXR) mediated signaling 114, 115. The endogenous ligands of the PPARs α, β/δ and γ are ranging from free fatty acids to various eicosanoids such as prostaglandins, leukotrienes and mono‐hydroxylated fatty acids 116. The RXR was described to be activated by 9‐*cis*‐retinoic acid (9CRA) 117, as well as the newly found endogenous relevant ligand 9‐*cis*‐13, 14‐dihydro‐retinoic acid/9CDHRA 118. It is debated whether 9CRA occurs endogenously 119. Currently, 9CRA is considered mainly as a ligand that is present after high non‐physiological and non‐nutritional relevant vitamin A intake, leaving 9CDHRA as the principal endogenous and the nutritional relevant RXR ligand. PPAR ligands are mainly food derived 116, while the nutritional precursors of the endogenous RXR ligand 9CDHRA were not yet identified. The PPAR‐regulatory pathway of BCO1/2 expression and further carotenoid bioactivation is thus controlled by the amount and fractional distribution of lipids present in the food matrix. In addition to genomic regulation of BCO1/2 expression, carotenoid cleavage can also be modulated by inhibitory effects of lutein on β‐carotene cleavage 120. This indicated that not just the individual carotenoid concentration is of relevance to bioactivation towards retinoic acid and further transcriptomic regulation, but also the concentration of carotenoids inhibiting this metabolic step, as well as their concentration relative to β‐carotene. The consequences of BCO1/2 mediated regulation of retinoic acid synthesis and further transcriptional signaling by additional factors and its consequences for our health will be discussed later (chapter 7), highlighting the special importance of BCO1/2 on explaining interindividual variability, likely related to the beneficial health effects of carotenoids.

### 3.3 Colonic fermentation as an interindividual source

To date, it is unclear to what extent the microbiota contributes to carotenoid metabolism, and whether carotenoids/ their metabolites can be taken up in the colon. It is known that a large proportion of carotenoids reaches the colon, as only 5–50% are absorbed in the small intestine. It is also known that carotenoids are partly bioaccessible in the colon 121. However, only 10–50% of the carotenoids remain intact after fermentation, while the remainder reacts to unknown compounds 121, 122, 123. This was supported by carotenoid standards as the only fermentation source in vitro, as >98% losses for β‐carotene and zeaxanthin were reported 123.

Very little is known on carotenoid interaction with the microbiota 124. Unlike polyphenols, which are heavily metabolized, no carotenoid degradation products/bacterial metabolites have been identified. In general, bacteria in the colon are able to deglycosylate, hydrolyse, deglucuronidate, demethylate, and cause ring‐fission in some molecules, among other 46, 125. However, in germ‐free rats, higher carotenoid utilization (of α‐ and β‐carotene) as measured by their liver levels, has been reported compared to rats with intact microbiota 45. It was suggested that indirect effects, such as decreased intestinal transit time and an altered bile pool in the absence of bacteria could have played a role, though a reduced level of bacterial breakdown products and more remaining native compounds could have been involved. In support of a potential absorption of carotenoids in the colon, a study in mice found BCO1 to be expressed in many cells including mucosal, glandular cells in the stomach, small intestine, and the colon 126. BCO2 is known to be expressed in almost all cell types known to express BCO1. However, BCO2 was not found in the colon, suggesting that only symmetric cleavage of carotenoids may happen in the mucosal cells in the colon.

In a previous study, β‐carotene uptake into human exfoliated epithelial cells of the colon, separated from feces, has been demonstrated 127. Following the consumption of β‐carotene rich spirulina, the concentration of β‐carotene in the cells increased approximately 3‐fold, demonstrating colonic cellular presence. However, this may have occurred not necessarily through direct cellular uptake via colonocytes, as carotenoids could have been absorbed via the small intestine and then distributed via the circulatory system to the colonocytes. Furthermore, the same constituents known to enhance carotenoid bioavailability, namely bile salts, emulsifiers such as lecithin 128, 129, enhanced colonic cellular uptake. Though carotenoids can be taken up by colonic derived Caco‐2 cells, direct colonic uptake is not easy to prove, and studies so far have not suggested a strong correlation between dietary intake of carotenoids and colon concentrations 130. Oshima et al. 131 investigated colonic absorption and distribution of lycopene in rats with or without a colostomy at mid colon that diverted the fecal stream but without resection of the distal colon. In rats given intragastric treatment, lycopene was found in the mucosa in the proximal colon and in the distal colon, also of the colostomized rats, whose distal colon was isolated from the faecal stream, indicating that lycopene may be transported via the blood into the colon. Moreover, lycopene reached the liver to an appreciable extent even when administered into the isolated distal colon, indicating that absorption is possible from the distal colon in rats.

Taken together, these results indicate that carotenoid absorption from the colon could be relevant and contribute to interindividual variation in carotenoid bioavailability, depending on the food matrix and microbiota. Furthermore, as faecal transplants have shown to be able to trigger obesity, at least in animal models 132, and obese subjects having generally lower concentrations of circulating carotenoids (Table 1), a potential direct or indirect link between the microbiota and carotenoid tissue levels may exist. In a study with atherosclerotic subjects, patients showed a metagenome with reduced phytoene‐dehydrogenase and lower β‐carotene serum levels compared to healthy controls, which was associated with a higher level of Collinsella spp. in diseased subjects 133, highlighting the potential role of the microbiota.

### 3.4 Diseases and medical intervention effecting the intestine and colon

Any condition reducing the intestinal mucosal surface area can be expected to reduce carotenoid absorption. As most studies do not directly measure carotenoid absorption efficiency but rather look at blood carotenoid levels (or a plasma fraction), it is important to distinguish between direct effects on carotenoid absorption (*i.e*. through reduced mucosal surface area or limited transport capacity) and indirect effects (through dietary adaptations, *e.g*. high fiber or low fat diet). This is usually achieved by controlling for carotenoid dietary intake.

A study with 20 Crohn's disease patients reported lower fasting blood carotenoid concentrations, independent of dietary intake 134, suggesting that malabsorption affected carotenoid uptake, though increased turnover rate and colonic losses via e.g. bleeding could not be excluded. Similar results were obtained by Geerling et al. 135 in a study with 32 Crohn's disease patients and Genser et al. 136 with 24 patients. Crohn's disease usually affects the ileum but only three of the 20 patients in the study had ileal inflammation, indicating the importance of the colonic mucosal integrity for carotenoid absorption. Patients undergoing bariatric surgery (Roux‐en‐Y gastric bypass and biliopancreatic diversion) also displayed lower blood carotenoid levels 137. Since fruit and vegetable consumption was apparently normal, the effect was attributed to malabsorption due to reduced mucosal surface area and also due to limited capacity of transport related to decreased lipoprotein concentration. Also reduced gastric digestion (via gastric lipase, or mechanic dispersion), could have played a role, as could have biliopancreatic diversion, affecting bile and pancreatic enzyme concentrations in the gut. In another study, subjects with Celiac disease and Crohn's disease (*n*  =  22) showed significantly 37% decreased levels of macular carotenoids compared to controls (*n*  =  25 138.

Short bowel syndrome, usually due to large resections of the small intestine to treat pathologies such as Crohn's disease or gastrointestinal tumors, have also been associated with carotenoid malabsorption. Edes *et al*. 139 reported undetectable β‐carotene blood levels following supplementation, despite adequate fat absorption, in a patient with extensive small intestinal resection (serum vitamin A levels appeared normal). Perhaps carotenoid absorption occurred in a more limited section of the intestine, or absorbed β‐carotene was fully converted to vitamin A. Luo *et al*. 140 reported no increase in blood carotenoid levels in subjects with short bowel syndrome undergoing intestinal rehabilitation, despite a 12‐week‐long supplementation with β‐carotene, lutein and lycopene. This was attributed to low fat absorption (about 30 versus >95% in healthy subjects) in these patients. However, no estimates of the contribution of the colon to the observed differences in absorption efficiencies were reported. Therefore, it is uncertain if it is the disease affecting the lower gut, the limited length of residual ileum, the presence or absence of the colon, the patient's lifestyle, or a combination that results in low plasma carotenoids. Similar low levels were observed in 63 patients with total gastrectomy 141, possibly due to duodenal bypass and short interposition of a small intestine loop.

Intestinal parasites and bacterial overgrowth can also damage mucosal cells and result in increased permeability and decreased absorption of nutrients. In Indonesian children receiving red sweet potato, serum retinol concentrations increased to a greater extent when children infected with intestinal helminths were dewormed, than when the intensity of infection was high 142, though the effect may have been also due to improved fat absorption. In tropical countries, also enteropathies, resulting in inflamed epithelium and reduced surface available for absorption, are likely to contribute to low carotenoid and vitamin A status 143.

## 4 Host factors influencing intracellular transport and transport to the liver

### 4.1 Intracellular transport within the enterocyte

After their uptake at the apical side of the enterocyte by membrane proteins, which are involved in the uptake of other liposoluble micronutrients, e.g. vitamin E/D 144, carotenoids have to cross the aqueous environment of the cell to reach its basolateral side. As carotenoids are very hydrophobic 21 it is assumed that they need to be associated with intracellular proteins to move through this medium 36. Though candidate proteins have been suggested, limited evidence of their involvement is available yet. A first one is human retinal lutein‐binding protein 145, as it shows a good cross‐reactivity with antibodies raised against carotenoid‐binding protein, which has been shown to transport carotenoids in the midgut cytosol of the silkworm *Bombyx mori* 146. However, its expression in the enterocyte should be verified. Other candidates could be the enterocyte FABPs (FABP2/I‐FABP and FABP1/L‐FABP) that allow the transport of various lipids. Finally, it can be hypothesized that the main enzyme responsible for carotenoid cleavage in the enterocyte, i.e. BCO1 39, 96, could also be involved, as it attracts and binds carotenoids for further cleavage, and it may also function as a non‐identified but predicted selective carotenoid‐transporter. The involvement of some of these candidate proteins in carotenoid transport within the enterocyte is supported by studies that have observed associations between SNPs in genes encoding these proteins and carotenoid status or bioavailability. This is the case for *FABP* and lycopene 103 and *BCO1* and β‐carotene 32, though this second association can also be due to the catalytic activity of this protein. Functional studies employing cell cultures or transgenic mice should be performed to identify the respective proteins. Nevertheless, it can be hypothesized that variations in genes encoding proteins involved in the transport of carotenoids within the enterocyte contribute to the observed interindividual variability in carotenoid bioavailability.

The previously described interaction of lutein and β‐carotene was not investigated further in detail, but it was predicted also to be of relevance regarding mutual interferences during absorption 33, 120, 147. A different fractional absorption efficacy was also suggested for *cis*‐isomers of lycopene 20, 148, 149, 150. Unfortunately, for lutein and β‐carotene as well as for lycopene and β‐carotene *cis*‐isomers, the mechanism of this altered transport efficiency was not examined further, but it appears to have an important physiological importance due to the different and possibly augmented health beneficial effects of especially 9‐*cis‐*β‐carotene versus all‐trans‐ β‐carotene, at least in respect to atherosclerosis 151.

### 4.2 Secretion at the basolateral and apical side of the enterocyte

During the postprandial period following the intake of a meal providing carotenoids, the latter are recovered in chylomicrons and their remnants, circulating in the blood 14, 32, 73. This allows physiologists to conclude that carotenoids are incorporated into chylomicrons within the enterocyte, then secreted into the lymph, and finally transported to the blood. Two observations support this paradigm. First, studies on Caco‐2 cell monolayers, an acknowledged model of the human intestinal epithelium, have shown that carotenoids added to the apical side of these cells are recovered in the lipoprotein chylomicron‐rich fraction secreted at the basolateral side 152, 153. Second, clinical studies have shown associations between SNPs in *MTP*, involved in chylomicron formation within the enterocyte, and *APOB (*the main chylomicron apoprotein), and carotenoid bioavailability 14, 32, 73. Secretion via chylomicrons implies that polymorphisms of genes involved in chylomicron formation, such as those involved in cholesterol biosynthesis, may potentially have a role in explaining inter‐individual variation in carotenoid uptake or processing, as has been suggested for patients with hypercholesterolemia 154.

Although it is acknowledged that a significant fraction of newly absorbed carotenoids is secreted by the enterocyte via chylomicrons, it should be noted that another fraction is metabolized within the intestinal cell. The size of this fraction depends on several factors such as the carotenoid species and the vitamin A status, affecting provitamin A carotenoid absorption and cleavage 108. As stated above, BCO1 and BCO2 are responsible for this mechanism. Their action results in several carotenoid metabolites, e.g. retinal, apo‐carotenals etc. 155, which may not share a fate similar to that of the parent molecules, and thus are not necessarily incorporated into chylomicrons. As at least some of these metabolites are water soluble (logP‐values around 5, such as for retinoic acid ‐ 4.4, http://www.drugbank.ca/drugs/DB00982), it can be hypothesized that they may be secreted to the portal vein and then reach the liver.

Another pathway involved in carotenoid secretion at the basolateral side of the enterocyte may be via APOA1. This involves the membrane protein ABCA1, responsible for the lipid transfer from this membrane to APOA1/HDL in the lymph. Though it was shown that ABCA1 is not involved in the efflux of carotenoids to HDL at the basolateral side of Caco‐2 cells 153, a recent study demonstrated that a fraction of carotenoids, at least the xanthophylls, is transferred via ABCA1 to APOA1, not directly to HDL 156.

Thus, the complex mechanisms that are involved in the secretion of carotenoids, and of their metabolites at the basolateral side of the enterocyte involve several genes and are likely to be modulated by genetic variations affecting the expression or activity of the proteins encoded by these genes. It was thus hypothesized that SNPs in these genes correlate with interindividual variability of carotenoid bioavailability. This hypothesis was supported by results of three recent human clinical studies. These have shown that SNPs in *MTP* and in *APOB*, involved in the APOB dependent pathway, as well as SNPs in *ABCA1*, involved in the APOA1 dependent pathway, are associated with lutein 14, lycopene 73, and β‐carotene 32 bioavailability. SNPs in *APOB* were associated with β‐carotene concentrations while SNPs in apolipoprotein A4 (*APOA4)* and *APOB* were associated with lycopene concentrations 103. These SNPs explained differences in e.g. β‐carotene plasma concentrations by up to 50%.

Finally, carotenoids may also be re‐excreted via the apical side into the gut lumen. Results from a human intervention trial (with tomato puree) suggested that the *ABCB1* gene plays a key role in lycopene transport, possibly by effluxing a fraction of the absorbed lycopene back into the intestinal lumen 73. This hypothesis needs to be examined further.

### 4.3 Postprandial chylomicron transport and blood plasma appearance

It is believed that most newly‐absorbed carotenoids are postprandially secreted in chylomicrons, and that the role of chylomicrons, among other, is to carry carotenoids and their lipophilic metabolites from the intestine to the liver. During their transport, chylomicron triglycerides undergo hydrolysis by LPL, resulting in the generation of smaller chylomicrons termed chylomicron remnants. After their uptake by the liver, a fraction of carotenoids appears to be stored in the liver, another one is metabolized (e.g. into vitamin A for the provitamin A carotenoids). The remaining fraction is re‐secreted into the blood within VLDL. VLDL, via their metabolism into LDL, are thought to be responsible for the further tissue distribution of carotenoids. Due to their hydrophobicity, it is thought that carotenoids stay located within the core of the chylomicron(remnant)s during their transport in blood 157. Thus, it is hypothesized that chylomicron carotenoids i) are not significantly transferred to other circulating lipoproteins (VLDL, LDL, HDL), and ii) they are not significantly transferred to tissues. However, an in vitro study has suggested that this assumption needs to be revisited because an exchange of carotenoids between VLDL and HDL was found 158.

Although it is possible that some chylomicron carotenoids can be transferred to other lipoprotein classes or to tissues during lipoprotein metabolism, it is assumed that this transfer is rather limited. Thus, the postprandial blood metabolism of carotenoids embedded in chylomicrons is closely related to lipoprotein metabolism. The metabolism of chylomicrons involves several proteins, starting with the apolipoproteins that are associated with these lipoparticles during their synthesis, i.e. APOB48 and APOA1, followed by the apoproteins that are transferred from other lipoprotein classes during chylomicron blood transport, e.g. APOE, and ending with enzymes that transfer or hydrolyse chylomicron lipids, e.g. cholesterol ester transfer protein (CETP) and LPL. Again, it is likely, though not yet demonstrated in humans, that some carotenoids, i.e. the less hydrophobic xanthophylls, can transfer from chylomicrons to other lipoproteins. Furthermore, in vitro data have suggested that *CETP* and *LCAT* (lecithin cholesterol acyl transferase) can be involved in this transfer 158.

Any variability of affinity of the above‐described transporters/proteins involved in chylomicron metabolism would alter carotenoid kinetics. However, only few human studies have examined these, including studies on lutein and β‐carotene 159, lycopene 160, and also retinyl esters 154. In the latter study, a 7‐compartment model demonstrated a saturable absorption process, in support of the uptake mostly via transporters. Variability of absorption was similar over the range of dosing (10‐120 mg), with a relative standard deviation (RSD) of ca. 50%. In an intervention study by Kostic et al. 159, adult subjects were given single equimolar doses (0.5 μmol/kg body weight) of lutein and/or β‐carotene solubilized in oil. Absorption had an RSD of 43 and 68%, respectively. A single peak of mean serum lutein concentration at 16 h was found, while for β‐carotene a small initial peak appeared at 6 h, and a second peak at around 32 h. The first peak was assumed to be chylomicron‐borne, the second peak was believed to represent newly absorbed β‐carotene from the liver circulating as VLDL/HDL 161, whereas the intermediate peak for lutein was unexplained. This suggests different mechanisms for the distribution of the two carotenoids, leading to a different time‐course of serum peaks, in line with an altered transfer between lipoproteins compared to carotenes.

It is unclear whether any differences in serum carotenoids described in the literature are related to any of the above proteins involved in uptake, transport and chylomicron metabolism, but it can be hypothesized. A variety of apolipoprotein polymorphisms were studied regarding concentrations of several carotenoids in children (*n =* 447), in a sample of the Stanislas Study. Lower concentrations of lutein/zeaxanthin (19%), β‐cryptoxanthin (51%), α‐carotene (55%) and β‐carotene (47%) were found in children expressing the S447X allele versus the S447S allele of the *LPL* gene 162, though no other correlations were found. In another study 163, human fasting concentrations of α‐ and β‐carotene were associated with genetic variants in *FABP* and *LIPC*, while α‐ and γ‐ tocopherol were influenced also by *APOC3* (a component of LDL), *CETP*, and *MTP* (required for lipoprotein assembly), indicating that these may be involved also in carotenoid metabolism. In an earlier study, serum concentrations of carotenoids (Table 4) were associated with SNPs in *APOB* and *APOA4* 103.

In addition to these proteins, other factors may play a role (Table 1). Brady et al. investigated the association between serum carotenoids and physiological and life‐style factors. Lower serum levels of several carotenes and xanthophylls were associated with being male (perhaps related to lower fruit/vegetable intake), smoker, of younger age, having lower non‐HDL cholesterol, higher alcohol consumption and higher body mass; only serum lycopene was not associated with these factors but with age 164. Age also showed to be significantly associated with chylomicron response of lycopene 165, but not with other carotenoids. However, the underlying mechanisms of these associations are unclear. It can be speculated that all factors are related to dietary pattern, though a higher body mass and a higher amount of adipose tissue may result in increased carotenoid storage in adipocytes, while smoking may increase the turnover of carotenoids due to enhanced oxidative stress (Table 1, 26). Similarly, in the SU.VI.MAX study (*n*>12 000 participants), it was found that β‐carotene plasma levels correlated (negatively) with smoking status, blood triglycerides, alcohol consumption and age. Again, females had higher β‐carotene serum levels than men (Table 1). Menstrual cycle also showed to influence plasma carotenoids. In an intervention trial with nine women consuming standardized diets for two cycles, carotenoid plasma concentration was usually lower in the earlier follicular phase compared to the late follicular phase and in part higher than in the luteal phase, possibly due to hormonal influences on the blood concentration of lipoproteins as carotenoid carriers 166. In a larger study, higher serum retinol levels were associated with higher serum estradiol and testosterone levels during the menstrual cycle 167.

## 5 Further transport and biodistribution to potential target tissues

### 5.1 Introduction

Carotenoids are transported in the blood stream associated with lipoproteins, where carotenes dominate carotenoid pattern in the LDL fraction and xanthophylls are almost equally distributed between LDL and HDL 168. Especially the potential exchange of xanthophylls between lipoproteins is important in this context and may depend on the activity of CETP and LCAT 158. Consequently, changes of the lipoprotein pattern, due to external or host‐related factors, may modulate tissue distribution of carotenoids 169. At the site of the target tissue, selective uptake systems may be operative to accumulate particular carotenoids, which are further transported to specific cells of the tissue; or within a cell, directed to subcellular compartments. Uptake might be hindered by tissue barriers (e.g. the blood–brain barrier), permeable only for certain compounds, though the lipophilic carotenoids would be expected to pass. Also, due to their lipophilicity, their volume of distribution (V_D_) in the body is quite large 170, and plasma concentrations will only to some extent reflect tissue levels. Thus, plasma concentrations are expected to be influenced if the V_D_ is altered, which may explain lower circulating carotenoid levels in obese subjects (Table 3). Consequently, this limits measuring plasma carotenoids as the most suitable marker of body status, and assessing additional compartments, such as following biopsies, or estimating various pools following isotopically labelled carotenoids, may constitute alternatives, though being more invasive or costly 19, 171. Unfortunately, only little is known about host related factors such as genetic makeup (e.g. SNPs) or other individual determinants and their impact on carotenoid tissue distribution.

### 5.2 Liver

Data from animal and human studies provide evidence that the hepatic tissue is a major site of carotenoid accumulation and metabolism 172, 173, 174, 175. β‐Carotene and lycopene e.g. are found in the nmol range per gram wet tissue, however, individual values widely vary (Table 5). Carotenoids travel with lipoproteins and the liver is a central hub for lipoprotein assembling and release. Hepatic endocytosis of chylomicron remnants, which contain newly absorbed carotenoids, depends on the interaction of the APOE protein with the membrane receptor LRP1 or to some extent with the LDL‐receptor. Also involved in the uptake mechanism is hepatic LPL. Carotenoids remain in the liver for storage, alternatively, they are secreted with VLDLs, which are further processed to LDLs. Mechanisms involved in the coordination of storage, cleavage and secretion are however not known yet. It is likely that the regulation of the specific cleaving enzymes plays a major role in carotenoid plasma levels.

**Table 5:** Carotenoid levels in liver and adipose tissue (nmol/g wet weight)

Tissue	nmol/g wet tissue (range), or ± SD			Reference
	β‐carotene	Lycopene	Lutein/zeaxanthin	
Liver	0.98	1.31	0.29	172
	(0.21‐3.94)	(0.16‐10.3)	(0.10‐0.66)	
Liver	4.41	5.74	3.22	173
	(0‐19.4)	(0‐20.7)	(0‐12.2)	
Liver	3.02	1.28	n.m.	175
	(0.16‐8.62)	(0.1‐4.08)		
Liver	15.06	25.46	2.94	174
	(9.1‐24.8)	(10.2‐55.1)	(0.2‐5.8)	
Mean across study	5.9 ± 6.3	8.4 ± 11.5	2.2 ± 1.6	
Total carotenoids in liver^a^ (nmol)	9.2	13.2	3.4	
Adipose	0.2	0.7	0.79	172
	(0.05‐2.37)	(0.02‐3.7)	(0.29‐2.7)	
Adipose	0.38	0.2	n.m.	175
	(0‐1.05)	(0‐0.51)		
Adipose	n.m.	0.23 ± 0.16	n.m.	301
Adipose	0.37 ± 0.34	0.32 ± 0.35	1.58 ± 0.93	188
Mean across study	0.32 ± 0.10	0.36 ± 0.23	1.19 ± 0.56	
Total carotenoid in adipose tissue^b^	4.4	5.1	16.6	

Data shows mean values and range or standard deviation.

n.m.: not measured

a assuming an average adult liver mass of 1561 g 302

b Assuming an average weight of body fat in non‐obese adults of 14 kg 303

As noted in earlier publications, tissues such as the liver (or testes and adrenals) which possess a large number of LDL receptors, exhibit high levels of carotenoids. On the other hand, lipids from circulating HDL are taken up by this organ, too. The central role of the liver in lipid metabolism makes it likely that individual differences (polymorphisms) in proteins affecting this process can influence carotenoid distribution. SNPs in LDL receptors may play a role, as they are critical for the endocytosis of the remnant chylomicron particle into liver hepatocytes, influenced by the binding of APOE to the surface of chylomicrons 176, and may therefore be suspected to play a role in carotenoid distribution. Though carotenoid levels in plasma have been associated with genetic polymorphisms in genes related to lipid metabolism 103, an impact on tissue distribution or uptake has not been proven so far in humans. By contrast, mice expressing APOE4 as compared to APOE3 had lower levels of β‐carotene in the bloodstream and lower levels of β‐carotene and lutein in adipose tissue 177, while hepatic expression of BCO1/2 was significantly higher, suggesting a correlation of both factors.

In addition to the conversion of provitamin A carotenoids into retinal by BCO1, the liver is also a central tissue for xenobiotic metabolism, mediated by an array of phase I/II enzymes. Especially the metabolism of carotenoids by cytochrome P450‐dependent monooxygenases has been topic of research, and an active hepatic P450 dependent metabolism was shown for several carotenoids 178, 179. Several metabolites have been detected 180 and thus a great deal of *P450* related genes expressed in the liver including *CYPs 3A4, 2C9, 2C8, 2E1*, and *1A2*, and to a lesser extent *2A6, 2D6, 2B6, 2C19*, plus the extrahepatically expressed *CYPs 2J2, 1A1*, and *1B1*, are expected to potentially influence carotenoid tissue levels 181. Phase I and II enzymes are usually inducible and respond to internal and external challenges (stress) a host is exposed to. Thus, in addition to genetic factors, also external factors can influence the extent of metabolism and metabolic pattern. A number of different polymorphisms involved in phase I/II enzymes in humans have already been revealed. They individually affect the metabolism of drugs or endogenous compounds. Little is known with respect to carotenoids, but it was shown that a polymorphism regarding *CYP26B1* (rs2241057) influences the degradation of retinoic acid, and is likely related to the risk of Crohn's disease 182 and atherosclerosis 183.

### 5.3 Adipose tissue

Adipose tissue and in particular the lipid fraction of adipocytes is an important site of carotenoid accumulation 172, 184, 185. Vitamin A is stored in adipose tissue primarily as unesterified retinol 186, 187. Concentrations of carotenoids in adipose tissue have been reported by several groups 188, 189, 190 (Table 5). According to these studies, concentrations of β‐carotene, β‐cryptoxanthin, lycopene and lutein/zeaxanthin were comparable in variation and concentration to their plasma concentration. Although concentrations of carotenoids per g tissue are higher in some other organs, adipose tissue contains the highest total amounts, and is assumed to contribute to carotenoid storage. Unfortunately, knowledge is scarce on the mechanisms involved in the regulation of carotenoid uptake/release in this tissue.

LPL, expressed in adipose‐ and other tissues, is the primary enzyme responsible for triacylglycerol lipolysis, provided by chylomicron‐ and VLDL transport vehicles for carotenoids, and implicated in fatty acid uptake. Thus, it may also play a role in adipocyte carotenoid uptake. Cell culture studies suggest that CD36 is involved in the uptake of lycopene and lutein by adipocytes 88. Hormone sensitive lipases are implicated in the release of retinol from storage tissue due to cleavage of retinyl esters, and may aid in releasing retinol by hydrolyzing triglycerides of the intracellular fat droplets 191. However, their impact on carotenoid release from adipose compartments is unclear. Retinol binding protein 4 (RBP4) is synthesized by adipocytes as a signaling molecule. It was shown to coordinate bidirectional retinol uptake in adipose tissue together with its membrane receptor STRA6 (stimulated by retinoic acid gene 6) 192. Retinol‐loaded holo‐RBP4 blocked adipocyte differentiation by activating RARα, while retinol‐free apo‐RBP4 triggered retinol efflux, resulting in reduced cellular retinoids and RARα mediated transcription and enhanced adipogenesis 192.

Several host related factors have been identified in EURAMIC (European multicenter case‐control study on antioxidants, myocardial infarction and breast cancer), scrutinizing correlations of carotenoid levels in adipose tissue 193. In another trial, obesity was associated with carotenoid levels in adipose tissue, though it is not quite clear whether this could also be due to decreased dietary intake. A correlation was also observed between higher alcohol intake and lower levels of β‐carotene and lycopene in adipose tissue of men and women, respectively. Although the concentration of β‐carotene in the adipocytes of obese subjects was lower compared to non‐obese, the total amount of β‐carotene in all lipid stores was similar 194. Whether this implies a regulation of total lipid body stores is still controversial. Regarding alcohol consumption, lower intake, altered liver‐metabolism, decreased small intestinal uptake, or enhanced consumption due to oxidative stress may play a role. In this context, the activity of β‐carotene metabolizing enzymes is likely important and genetic differences but also alcohol intake is expected to affect a balanced distribution in adipose tissues.

Mice in which *BCO1* is deleted and receiving marginal vitamin A sufficient diet with β‐carotene, accumulate β‐carotene in adipose tissue 102. This illustrates the role of adipose tissue as a storage tissue for lipophilic compounds such as carotenoids. Recently, in yellow rabbits, a triplet deletion was identified in the *BCO2* gene, resulting in the absence of an asparagine in BCO2. This was suggested to cause accumulation of carotenoids in adipose tissue 195. This agrees with an earlier finding in sheep, where a *BCO2* mutation was found to be tightly associated with white adipose tissue carotenoid accumulation 196, and in bovines where *BCO2* mutations were associated with carotenoid accumulation in adipose tissue and milk 197, 198. These observations were lately confirmed by *BCO2* inactivation in sheep using CRISPR/Cas9 technology, resulting in yellow fat, establishing a causal relationship between BCO2 activity and carotenoid accumulation in white adipose tissue 199. Together, these findings not only illustrate the importance of white adipose tissue as a carotenoid storage organ, but also show whole body physiological regulation of carotenoid homeostasis, posing another layer of complexity on understanding inter‐individual variation (see also 6.4). This is exemplified by sex specific responses resulting from β‐carotene accumulation in white adipose tissue, which showed that 4970 genes were affected in WT female mice, while only 407 were affected in male mice 200, with the majority of the commonly affected genes (141 out of 144) showing a strong negative, rather than positive, correlation of expression between males and females. This negative correlation was also seen in *BCO1* knockout mice, although the number of genes affected were more similar between the two sexes (1522 gene in females and 1202 in males, 33 overlapping) 201. In both WT and *BCO1* knockout mice, only a minority of genes is commonly affected by β‐carotene in females and males. Strikingly, the opposite regulation of genes in response to β‐carotene exposure was also prominent in the lung of *BCO1* knockout mice, but this was not seen in WT mice in this tissue 202. On the other hand, WT liver showed a strong positive correlation of β‐carotene responsive genes between males and females 201.

Adipose tissue is distributed over various depots in the body. Functional differences between depots exist and visceral adipose tissue is especially associated with adverse health effects. In a study aiming to identify differences between adipose tissue depots, it was found that many of the genes differentially expressed in subcutaneous and visceral adipose tissue were regulated by retinoic acid 203. The master regulator of adipogenesis, PPARG is functionally active as an obligatory dimer with RXRA, linking adipogenesis with vitamin A metabolism. Retinoic acids are generated from retinaldehyde in adipose tissue by aldehyde dehydrogenase 1 (ALDH1), though this is discussed controversially 187. Female mice with inactivated ALDH1A1 were resistant to high‐fat diet‐induced visceral adipose formation. This was not seen in male mice, while subcutaneous adipose tissue was reduced to the same extent in males and females 204. Together, this underlines a role for vitamin A metabolism in differential adipose tissue (i.e. visceral versus. subcutaneous) formation. It has been suggested that estrogen mediated suppression of ALDH1A2/3 mRNA expression is involved in differential retinoic acid formation between males and females 205. Although major gaps in our knowledge exist, genetic variation in uptake, storage and processing in adipose tissue of various carotenoids may influence adipose tissue distribution and functionality and associated health outcomes, and, if so, will likely do this in a sex dependent manner. Effects of carotenoids on adipose tissue biology have been reviewed recently 206, while effects of genetic ablation of genes encoding various retinoid metabolism enzymes, including BCO1 and ALDH1A1, but also RBP1, RBP3 and retinol saturase *(RESTAT)* on adiposity in mice are reviewed elsewhere 203.

### 5.4 Skin

The carotenoid pattern in human skin comprises carotenes and xanthophylls. Plasma levels of lycopene and less notably β‐carotene are correlated with their respective concentration in the skin 207. However, no such correlation was observed for lutein, zeaxanthin, and β‐cryptoxanthin, and thus skin measurements may not be representative of total carotenoid exposure or status. Carotenoids are not equally distributed in the different skin areas. Highest levels occur in skin of the forehead and in the palms of the hands and lower levels in dorsal skin, inside of the arm or back of hand. LDL‐receptors are expressed in human skin 208 and may play a role regarding selective uptake. Skin can be divided into epidermis and dermis with underlying subcutaneous adipose tissue. Blood vessels reach the dermis but not the epidermis, and different ways of how carotenoids may be transported and distributed to and within the different layers of our skin have been discussed 209. Subcutaneous tissue is a storage compartment for carotenoids and part of the balanced distribution system of carotenoids in adipose depots. Thus, host factors already mentioned above are expected to also play a role in carotenoid uptake and storage in the skin. There is some evidence that other host‐related factors influence carotenoid skin levels 210. On the long term, a carotenoid‐rich diet may increase carotenoid skin levels. However, smoking and alcohol intake induced a rapid decrease in carotenoid levels of the skin. Skin lycopene levels are sensitive to UV‐irradiation 211. Upon irradiation in vivo, lycopene concentration in the skin is significantly lowered. This effect is less pronounced with β‐carotene. Therefore, individual preferences regarding sun or UV exposure (tanning beds) would affect skin carotenoid concentrations.

### 5.5 Macula lutea

The macula lutea is a small yellow area of the retina. It is the region of maximum visual acuity and its yellow color is due to xanthophylls, mainly lutein, zeaxanthin and meso‐zeaxanathin, located in the cone axons of the Henle fiber layer. It was shown that macular pigment density was positively correlated with serum concentrations of lutein and zeaxanthin, and inversely correlated with serum oxidized low‐density lipoprotein 212. Consequently, host related oxidative stress conditions would impact the supply of the retina with oxo‐carotenoids. Absolute levels and the patterns of lutein and zeaxanthin differ within an individual retina sample 213. Large interindividual differences have also been described 214. In the center of the macula lutea, levels of lutein and zeaxanthin have been reported, with 2.4 and 3.4 pmol/mm², respectively. Much lower concentrations are found in peripheral areas; medial 0.22 pmol lutein/mm² and 0.14 pmol zeaxanthin/mm². Levels further decrease in outer circles while the ratio of lutein:zeaxanthin increases. Since carotenes are not present in the macula lutea, selective mechanisms of uptake must be operative.

It is likely that host related factors (individual differences) have an impact on the density of the macula pigment. In a 6 months intervention study with a lutein‐rich supplement the impact of genetic variances in four genes (*ABCG8, BCO1, CD36, and NPC1L1*) on lutein plasma levels and macular pigment optical density was evaluated 215. The results provide evidence that (as with plasma) retina levels, i.e. macula pigment optical density of lutein are affected by SNPs of *CD36* and *BCO1*. The TT variant at the *BCO1* rs7501331 locus was associated with a higher macula pigment optical density. Study subjects with GG at the *CD36* locus rs1761667 had a higher macula pigment optical density compared to those with an A allele, although the underlying mechanisms remain to be established. Compounds delivered to the retina must pass the blood–retinal barrier, provided by tight junctions between endothelial cells. This barrier is sensitive to inflammatory and oxidative damage often associated with hyperglycemia 216. HDLs as the transport vehicles of lutein and zeaxanthin have been discussed to play an important role in the transport of macular carotenoids 217. There is evidence that SR‐BI, a tissue receptor for HDL, plays a role in the delivery of carotenoids to this tissue 218.

It was further suggested that the delivery of macular carotenoids involves the inter‐photoreceptor‐retinoid binding protein. Retinoids and lutein/zeaxanthin have similar affinities to this protein, which facilitates the transfer of lipids across the inter‐photoreceptor space 219. GSTP1 has been identified as the macular binding protein for zeaxanthin/meso‐zeaxanthin in humans 220. It was shown that StARD3 acts as the lutein binding protein in the human macula 221. In‐vitro studies have proven the selectivity of both proteins with respect to either zeaxanthin or lutein. Equilibrium dissociation constants (KD values) for the complex of GSTP1 with zeaxanthin/meso‐zeaxanthin are in the range of 0.14 ‐ 0.19 μM while for lutein/β‐carotene they are >6‐fold higher. Contrarily, StARD3 exhibited a high affinity to lutein (KD ca. 0.59 μM), compared to zeaxanthin/meso‐zeaxanthin (KD‐values ca. 1.6 μM).

An alternative mechanism contributing to the selective enrichment of the macula carotenoids in primates has been suggested 222. The affinity of the human xanthophyll metabolizing BCO2 for lutein, zeaxanthin, and meso‐zeaxanthin is 10–40 fold lower than the affinity observed in mice, who do not accumulate these carotenoids in the retina. Thus, it has been speculated that ineffective cleavage of xanthophylls contributes to their accumulation in the macula lutea. BCO2 knockout mice, unlike WT mice, accumulate zeaxanthin in their retinas. Thus, genetic variances in the respective enzymes or transport proteins likely affect the accumulation, distribution, and metabolism of oxo‐carotenoids in the macula lutea.

### 5.6 Tissues relevant for cardiovascular diseases

Besides the adipose tissue, other tissues such as the pancreas and various cells of the vascular system, including endothelial cells and macrophages, are important targets of related health beneficial effects of carotenoids. In an older study, carotenoid levels in the pancreas were found to be comparable (1.8‐4.5 μg/g wet weight 223 or 4.5‐95 μg/g 224) to those in adipose tissue levels. The beneficial and risk preventive effects of carotenoids on atherosclerosis development were suggested to be mediated in macrophages and the endothelial cells of the blood vessels 225, 226. Unfortunately, a direct mechanistic connection of carotenoid levels in white blood cells and especially monocytes/macrophages, as well as endothelial cells and further induced biological effects was never examined and confirmed.

As carotenoids were described in relation to the prevention of T2D, a potential target organ is the pancreas as a major regulator for insulin and glucagon production and secretion. Recently, RAR‐ and RXR‐mediated signaling pathways were positively and negatively correlated with insulin and glucagon secretion 227, respectively. As pro‐vitamin A carotenoids are the major precursor for the physiological and nutritional ligands of these two receptor subclasses 228, a further genetic regulation related to carotenoids appears plausible, but has not yet fully confirmed and described. This plausible correlation to T2D, starting from beneficial regulatory pathways of retinoids as carotenoid metabolites via RAR‐ and RXR‐mediated signaling pathways seems also to be highly dependent on carotenoid accumulation for substrate availability 229.

### 5.7 Other tissues

Carotenoids occur in almost all human tissues. However, some were in the focus of research. Based on epidemiological studies, it was proposed that a frequent intake of tomato products rich in lycopene is associated with a decreased risk for prostate cancer 230, though it is unclear whether lycopene is selectively taken up in the prostate and which mechanisms may be relevant in this context. Levels of lycopene in prostate tissue have been reported around 1.7 ng/mg tissue following supplementation 231; similar to levels in adipose and liver (Table 5).

In the human brain carotenes and xanthophylls were detected and the latter accounted for about 70% of total carotenoids, with lutein and zeaxanthin dominating 232. Levels were different in different brain areas and ranged from 2.8 to 11.8 pmol/g for lutein, 1.8 to 9.2 pmol/g zeaxanthin, and 7.6 to 15.2 pmol/g β‐carotene. A supplementation study with rhesus monkeys has shown that the brain levels of lutein and zeaxanthin are significantly related to their levels in the macula lutea 233, and therefore, macular pigment density may be used as a surrogate biomarker of lutein and zeaxanthin in primate brain tissue. Analyses of human brain tissues revealed a relationship between StARD3 levels and the concentration of lutein 234, with strongest correlations observed in infant (versus. adult or centenarian) brains.

Colostrum contains significant amounts of α‐ and β‐carotene, lycopene, β‐cryptoxanthin, canthaxanthin, lutein and zeaxanthin, which are responsible for the yellow color. With ongoing lactation the content of carotenoids in mature milk declines, and the carotenoid pattern changes 235, being correlated with lower lipid content of breast milk 236. This could suggest that a temporal specific mechanism is involved in the transfer of carotenoids to human milk, though via which mechanism is not understood. On the other hand, breast adipose tissue carotenoid concentrations from tumor patients were significantly related to serum concentrations, and were highest for β‐cryptoxanthin (3.5 μmol/kg), β‐carotene (2.3 μmol/kg) and lutein (1.8 μmol/kg), not indicating a considerable change of patterns 237.

Kidney concentrations, as other organ carotenoid levels, appear more variable than plasma concentrations, and have been reported to be in the range of 0.2–12.7 nmol/g for total carotenoids 173. However, a significant correlation for total carotenoids was found between liver, kidney and lung, thus additional discriminations of patterns could not be concluded. Again, host related factors especially those regarding the genetic variations of the proteins mention above might have impact on carotenoid distribution in these tissues.

## 6 Host factors interacting with carotenoid storage and excretion pathways

### 6.1 Storage and turnover aspects

Very little is known on the metabolism of especially non‐provitamin A carotenoids in xenobiotic pathways and on the extent of degradation and elimination of the parent compounds 238. Lutein and β‐carotene may interact with each other during postprandial serum clearance when administered together. Both mutually enhancing and inhibiting actions have been observed in the limited number of volunteers studied. Interindividual differences in the response were studied after a high dose (0.5 μmol/kg body weight) of either or both carotenoids followed by blood sampling 159, 239. On average, the volunteers showed faster postprandial serum elimination (up to 120 h) of both carotenoids when given together, while subsequent elimination (up to 32 days) was unaffected by the other carotenoid. In line with this trial, the loss of liver vitamin A in rats dosed with β‐carotene was not affected by concomitant dosing with lutein; however, the initial storage was enhanced by smaller lutein doses and inhibited by larger doses 240. Effects during β‐carotene absorption appear more likely to explain the inhibitory actions of lutein, whereas the enhanced vitamin A storage following β‐carotene dosing by low concomitant doses of lutein is more difficult to explain, and may involve interactions other than at the step of absorption. However, it is apparent from this study that (at least in rats) lutein does not affect subsequent loss of hepatic or renal stores of vitamin A. Whether a similar phenomenon on initial retinol storage exists in humans, which could partially explain interindividual variation in body stores of carotenoids is not known.

Carotenoid kinetic aspects were determined in depletion studies of 70–80 days in females (18–42 years) with and without isotope dilution by Burri et al. 241. Following a carotenoid controlled diet, carotenoids were measured in blood plasma of 19 healthy adults, and half‐lives recorded. Lutein had the longest half‐life (76 ± 17 days), followed by α‐carotene, β‐cryptoxanthin, zeaxanthin, β‐carotene and lycopene (45 ± 7, 40 ± 5, 38 ± 7, 37 ± 5, and 27 ± 3 days, respectively), with lutein and lycopene differing significantly in half‐lives from other carotenoids. Other studies based on postprandial designs have reported other half‐lives, such as 2–3 days for lycopene and 5–7 days for β‐carotene 242, likely to reflect plasma exchange with deeper compartments, while the longer reported half‐lives would reflect losses from those deeper compartments. In the study by Burri et al., all carotenoids followed similar first order kinetic rates, indicating a small variation in plasma kinetics but clear differences in carotenoid half‐lives. Concentrations of all carotenoids were highly correlated. The differences in carotenoid half‐lives were unexplained, though differences in degradation by acting as antioxidants or transfer to deeper compartments were suspected. Half‐lives were unrelated to physical or demographic characteristics (BMI, energy metabolism, cholesterol and triglyceride levels, ethnicity, or age). However, the number of subjects was small and participants in this study rather homogenous. Thus, it is difficult to judge the effect of half‐lives on interindividual carotenoid variations regarding blood levels.

In a study by Shvetsov et al. 243, the shorter half‐life of lycopene was considered as an explanation for the higher intraindividual variability of plasma lycopene compared to other carotenoids (lutein, β‐carotene). In their study, plasma carotenoids of 381 women were measured repeatedly (4 times) with 4 month intervals, and intraindividual variability was approximately half of that of interindividual variability, except for lycopene, where it was equal. Similar higher intraindividual variability for lycopene was also found by Cooney et al. 244. The source of intra‐individual variation is unclear. Seasonal variations appeared to be low (below 3% in the study by Shvetsov, partly due to the constant climate of Hawaii). When adjusted for age, race, alcohol drinking, and tobacco smoking, intraclass correlation coefficients were 0.69, 0.45, and 0.74 for total plasma lutein, lycopene, and β‐carotene, respectively. Dietary factors, age, gender, ethnicity, geographic location, and season were employed as main factors to explain intraindividual variability. A slightly better correlation with increased age was also reported, for reasons unknown. Further studies need to investigate the potential effect of diet, life‐style and additional factors on carotenoid depletion rates.

Not much is known regarding carotenoid excretion pathways. Khachick et al. reported on polar metabolites of lycopene in humans 179, approximately 5% of an isotopically labelled β‐carotene (^14^C) dose was excreted in urine (70% in feces) during 12 d 245. The minor fraction excreted in urine likely represents polar metabolites. In rat hepatocytes, it was shown that astaxanthin could be metabolized into 3‐hydroxy‐4‐oxo‐β‐ionol, 3‐hydroxy‐4‐oxo‐β‐ionone, and their reduced forms, 3‐hydroxy‐4‐oxo‐7,8‐dihydro‐β‐ionol and 3‐hydroxy‐4‐oxo‐7,8‐dihydro‐β‐ionone, and that this was associated with induction of the cytochrome P450 enzyme (CYP3A4 as well as of CYP2B6) 180. Thus, alterations in P450 enzyme activity may influence carotenoid metabolism. In human keratinocyte cells, it was shown that various CYP enzymes metabolized all‐trans retinoic acid and cis‐isomers into water soluble products 246.

### 6.2 Disease conditions altering carotenoid turnover

Several clinical conditions related to carotenoid status affect carotenoid concentrations in human plasma, in addition to those involved in hampering carotenoid uptake (see chapter 3.4). However, it is often difficult to assess whether this happens by interference with carotenoid uptake, excretion and/or metabolism. In patients with renal failure, plasma β‐carotene levels increased 247. In the same study, decreased plasma levels of β‐carotene were observed in subjects with liver cirrhosis. Similarly, a study measuring carotenoids in tissues by needle biopsies showed much lower hepatic levels at all stages of liver disease 248. Another study examined β‐carotene plasma concentration in 53 Filipino children with cholestatic liver disease and found decreased concentrations in 45 patients 249. This suggests that liver diseases interfere with storage and excretion of carotenoids. In the same study, six children received a single dose of 10 mg/kg body weight of β‐carotene. No increased plasma levels were detectable in 5/6 children, pointing to a main effect of the disease on β‐carotene absorption rather than further metabolism (re‐distribution or excretion). This was explained by missing bile salt secretion and reduced solubilisation and cellular uptake. However, infections (e.g. helminths) could not be excluded as an additional factor.

The liver, via the bile, also plays an important part in the excretion of carotenoids back into the gut. As many transporters expressed in the intestine are also present in liver cells 250, it can be assumed that genetic differences in e.g. *SCARB1* and *CD36* do also influence biliary excretion, however, this constitutes a gap in our knowledge. In a study with the purpose of comparing plasma and biliary concentrations of carotenoids among controls and patients with biliary and pancreatic diseases, both plasma and bile concentrations of β‐carotene were significantly decreased in patients with bile duct stones, impairing biliary excretion. Moreover, the plasma/bile ratio was maintained as well as the correlation between them, and plasma β‐carotene decreased even more in patients with complete biliary obstruction, probably reflecting malabsorption due to limited carotenoid solubilisation in the gut. A tight correlation between plasma and bile β‐carotene still persisted in patients with pancreatic disease, confirming the role of plasma β‐carotene in determining bile concentrations 251. The authors concluded that carotenoids undergo, at least in part, biliary excretion, that biliary concentrations reflect plasma levels in both normal and pathologic states, and that a decreased biliary excretion does not increase plasma concentrations. The study thus mainly highlights the importance of the bile for carotenoid absorption and the consequent tight link between bile and plasma levels.

Obesity was associated with circulating plasma carotenoids in several studies (Table 1), however, the amount of carotenoids in the total adipose tissue has been found constant among obese and normal‐weight subjects 194. It is possible that a higher amount of adipose tissue with its high affinity to store carotenoids merely reduces the release of carotenoids into the bloodstream or enhances their uptake from the circulatory system, possibly via LDL receptors. As obesity is related to chronic inflammation, it cannot be excluded that upregulation of nuclear factor kappa‐B (NF‐κB) and nuclear factor erythroid‐derived 2 like 2 (NRF2) 252 is somehow related to a higher degradation rate of carotenoids, but this remains hypothetical. On the other hand, it is possible that the body may adapt to increased oxidative stress by upregulating circulating plasma antioxidants. For example, in a study with asthmatic women, higher levels of total carotenoids were found compared to non‐asthmatic control women 253.

An enhanced conversion of β‐carotene to retinol was suggested to explain significantly lower (by approximately 50%) serum levels of β‐carotene in hyperthyroid patients compared to those with hypo‐ and euthyroidism 254. However, the detailed mechanism remains unclear. Hypothyroidism also led to increased (2‐fold) β‐carotene absorption in this study, explaining the yellowing of the skin in these patients. Thyroid hormones may thus alter β‐carotene absorption, however an effect on its distribution cannot be ruled out.

### 6.3 Effect of vitamin A status and lifestyle on carotenoid status

It has been demonstrated that vitamin A status modulates β‐carotene absorption and cleavage (see chapter 3.2). A study examined the ability of deuterated retinol‐dilution to detect changes in the body pool size and status of vitamin A and the effect on the bioconversion of carotenoids to vitamin A 255. Changes were detected in the body pool size after 3 days. The bioconversion of dietary mixed plant food carotenoids varied inversely with vitamin A status, and improvements in status after intervention were strongly affected by total body stores of vitamin A, which can be explained by feed‐back mechanism of vitamin A status and BCO1, highlighting the relation of circulating pro‐vitamin A carotenoids and vitamin A status.

Chronic alcohol consumption may perturb vitamin A and carotenoid metabolism. A study in rats given vitamin A or β‐carotene examined the effect of chronic alcohol consumption on vitamin A status and found a decrease in hepatic vitamin A storage, which was not due to malabsorption of either retinyl acetate or β‐carotene, nor to altered activities of several enzymes involved in ethanol or vitamin A metabolism 256. However, other studies show inconsistent findings; studies suggested inhibition of BCO1/2 by ethanol 257.

### 6.4 BCO1/2 aspects with respect to carotenoid tissue levels

As for the intestine, disruption of BCO1 expression is well known to reduce vitamin A and increase β‐carotene concentration in tissues, identifying BCO1 as the major enzyme for vitamin A production and for carotenoid cleavage 258. The role of carotenoid oxygenases involved in the cleavage and storage of carotenoids is confirmed by other studies. For example, expression of BCO1 was documented by RNA blotting and immunostaining methods in a wide selection of human tissues 126, 259. In the study by Lindquist et al. in mice, BCO1 was expressed in virtually all tissues, and the same is assumed for humans. An animal study examined the effect of knockout BCO1/2 in mice given a controlled diet primarily providing β‐carotene 39. Accumulated levels of β‐carotene in serum, liver and lungs in BCO1^(−/−)^ and BCO1^(−/−)^/BCO2 ^(−/−)^ mice were found. BCO1^(−/−)^ mice showed 100 fold higher concentrations of β‐carotene in tissues compared to wildtype and BCO2^(−/−)^ mice, confirming the role of BCO1 as the major β‐carotene‐metabolizing enzyme. This does not negate a role for *BCO2*, since *BCO2* inactivation in sheep resulted in carotenoid accumulation in adipose tissue 199.

Another study in mice investigated the effect of gene expression induced by β‐carotene supplementation, knockout of BCO1, and differences in gender on β‐carotene levels in lungs, liver and inguinal white adipose tissue 200. Lungs were mainly affected by knockout, liver by knockout and gender, while the white adipose tissue was mainly affected by gender. Hardly any β‐carotene affected genes were in common in the three tissues, suggesting that changes in gene expression are primarily determined by tissue and gender.

β‐Carotene exposure increases β‐carotene concentration in the lung, but also the concentrations of retinol and retinyl esters 260. Inactivation of *BCO1* increases β‐carotene concentrations, and decreases retinyl ester concentrations in males and females, while retinol concentration are only decreased in females 261. Differential regulation of genes involved in vitamin A metabolism in the lung upon β‐carotene exposure, for example *LRAT* (conversion of retinol in retinyl esters) and *ADH7* (conversion of retinol into retinal) suggest that polymorphisms in these genes can also have a role in interindividual responses, which could be relevant because these genes can functionally determine retinoid sufficiency 260.

## 7 Bioactivation ‐ from carotenoids to nuclear hormone receptor ligands and further induced transcriptional signaling

The mechanisms of action of carotenoids regarding beneficial health effects comprises two major pathways: a) functioning as direct antioxidants via various pathways 262, which has recently been discussed controversially due of lack of sufficiently high concentrations to transmit these effects endogenously 252 and b) functioning as precursors of various oxidative cleavage products 263. These can further interact with various nuclear hormone receptors such as the RAR, RXR, PPARs, LXRs, FXR, NF‐κB, and NRF2 252, 264. This conversion was mainly demonstrated in cellular and mouse studies, while in humans merely individual steps of this activation cascade were confirmed 97, 98, 229, 265, 266.

Assuming that that carotenoids function mainly via precursors of nuclear hormone receptors implies that not the serum and organ levels of native carotenoids are of major importance, but mainly the conversion of these endogenous present carotenoids to oxidative cleavage products. This again highlights the importance of the two carotenoid oxygenases BCO1/2. When focussing on health related effects of carotenoids, the focus should be placed on correlating carotenoid intake with resulting endogenous carotenoid metabolite levels and their effects on nuclear hormone receptor mediated signaling, not merely on the concentrations of the native carotenoids, however, data on these are still scant. In this regard, the retinoic acid receptors (RARs), with the isotypes RARα, β and γ, are activated mainly by the provitamin A carotenoid metabolite all‐*trans*‐apo‐15´‐carotenoic acid (all‐*trans* retinoic acid 267). In addition, non‐well examined pathways with low affinity activation ligands such as all‐*trans*‐4‐oxo‐retinoic acid 268, all‐*trans*‐3,4‐didehydro retinoic acid 269 and all‐*trans*‐13,14‐dihydroretinoic acid activation were described 270. The activation of the RAR as well as the RXR (RXRα, β and γ) by 9CRA seems to be of non‐physiological relevance, due to low or even non‐existing transcriptional activation of both RARs and RXRs by relevant endogenous levels 118, 119.

Recently, in addition to β‐carotene functioning as the major precursors for nuclear hormone receptor ligands for RARs and RXRs, the acyclic carotenoid lycopene was described to transmit further RAR‐mediated signaling 97, 271. Alternatively, RXR‐ and PPAR‐mediated signaling was postulated to be mediated via lycopene oxidative metabolites. The detailed mechanisms including the actively involved lycopene oxidative metabolites remains still controversial but it is likely involving apo‐15´‐lycopenoids for further RXR‐mediated signaling 97 and apo‐10´‐lycopenoids for further PPAR‐mediated signaling 98. Future research should focus on correlating carotenoid intake with carotenoid levels in the individual, further metabolic conversion to bioactive oxidative carotenoid metabolites, and the identification and quantification of marker genes related to beneficial health effects.

## 8 Conclusion and perspective

**Figure 2:**
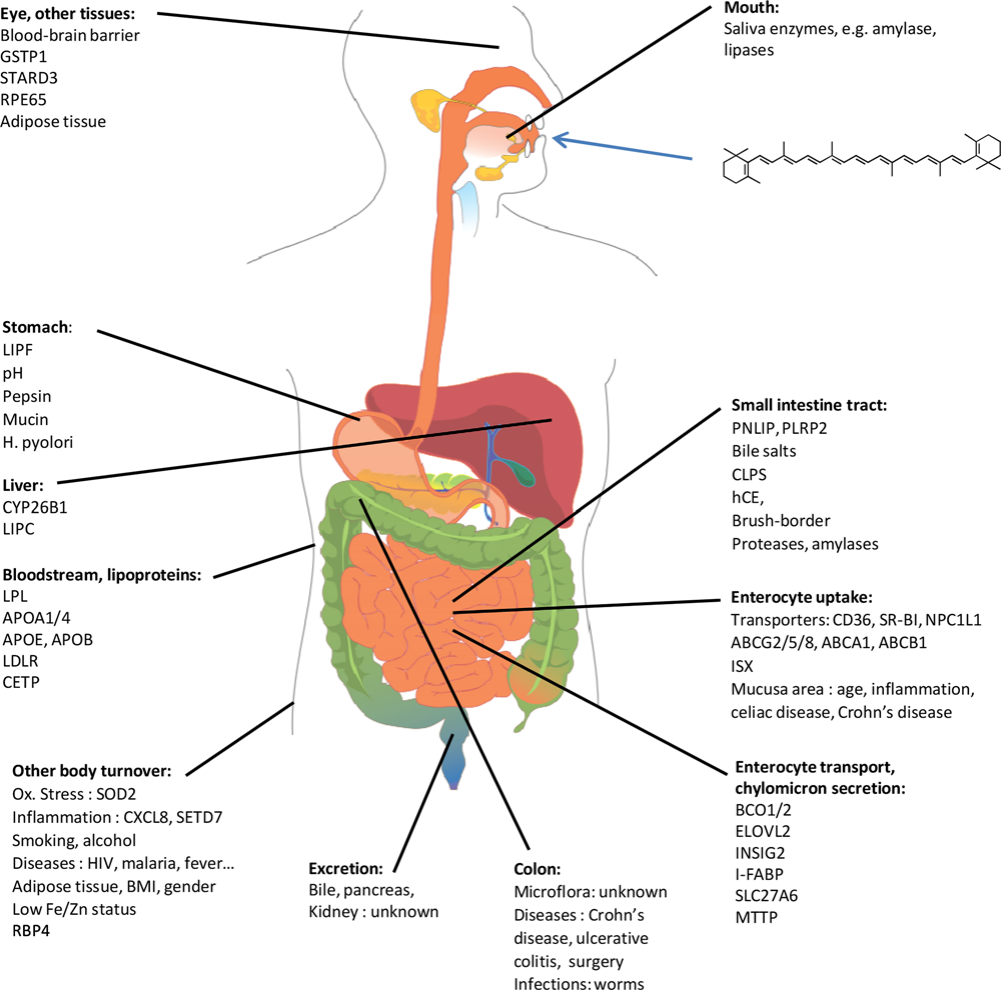
Overview of factors likely to contribute to interindividual variation of carotenoid bioavailability and thus tissue concentrations.

Interindividual carotenoid variability following intervention studies with dietary carotenoids has been investigated in a number of body compartments, including digesta, chylomicrons, blood, skin, and the retina. Additional variation has been observed in observational studies (Fig. 2). Variability in the carotenoid bioaccessible fraction is approximately half of that of plasma concentrations, approximately 20–30%. This may indicate that about half the variability may be explained by factors influencing carotenoid bioaccessibility, namely digestion enzyme concentrations, bile salts, and intestinal transit time. These are influenced by diseases (e.g. short bowel syndrome), the microbiota, and also gene expression related to enzymes (gastric lipase, PNLIP, CEL, CLPS). Factors influencing absorption also include parasites, such as hookworms, i.e. factors decreasing absorptive surface. Carotenoid absorption itself may be influenced by uptake transporters and associated SNPs (e.g. in the *CD36, NPC1L1*, and *SCARB1* genes). Intracellular transport and chylomicron or also HDL secretion (ABCA1) have been associated with ABCG5/8, FABP2, ELOVL2, INSIG2, SLC27A6, and MTP, and intracellular cleavage with BCO1, likely involving BCO2 for some carotenoids. Further biodistribution, affecting plasma levels, likely include LPL, LIPC, CETP, and APOA1, APOA4, APOE and APOB. Tissue incorporation is influenced by all these preceding processes, in addition to specific uptake transporters such as GSTP1, StARD3, and RPE65, in case of the retina. Many other SNPs, involved in inflammatory processes and certain diseases have been shown to correlate with carotenoid tissue levels (Table 4), though their exact role and contribution to variability remains to be elucidated. Hormones and gender play a role, as do possibly age and percentage of adipose tissue, though again the mechanisms are not comprehended. Several diseases have also been reported to influence carotenoid turnover and excretion, including hyperthyroidism and diseases of the liver and kidney, though the underlying mechanisms are not understood. While thus many of the potential candidates explaining interindividual variability, especially concerning cellular uptake and cleavage in the epithelium, have been determined, much less is known on their actual contribution to interindividual variability, and less is known on factors effecting intraindividual variability, which appears to be approximately half of interindividual variability according to some studies (Table 3). Influences of season and diet are most likely to contribute. In addition, we tried to emphasize connections between the individual carotenoid levels in the organisms and their bioactivation, resulting in oxidative carotenoid cleavage metabolites, mainly retinoids, and further mediation of transcriptional signaling of health related marker genes.

In the future, studies should aim at identifying additional SNPs related to carotenoid ADME parameters, to increase our knowledge on the contribution of genetic variations to interindividual variability. This may include SNPs in genes encoding for digestion enzymes and proteins involved in further tissue distribution, which so far have received limited attention. Furthermore, the connection between SNPs and health related marker genes should be in the focus of research to scrutinize carotenoid health protective effects. In addition, epigenetic factors and the microbiota are areas which until to date have been mostly overlooked. These will reveal new insights into explaining the variability of carotenoid concentrations in human tissues, but perhaps also explain varying biological responses to dietary intervention, opening the door to personalized nutrition and “food to health” strategies employing carotenoids.

*The authors have declared no conflict of interest*.
